# Immune cell and transcriptomic analysis of the human decidua in term and preterm parturition

**DOI:** 10.1093/molehr/gax038

**Published:** 2017-08-22

**Authors:** S.F. Rinaldi, S Makieva, P.T. Saunders, A.G. Rossi, J.E. Norman

**Affiliations:** 1 MRC Centre for Reproductive Health and Tommy's Centre for Maternal and Fetal Health, University of Edinburgh, Queen's Medical Research Institute, Edinburgh, UK; 2 MRC Centre for Inflammation Research, University of Edinburgh, Queen's Medical Research Institute, 47 Little France Crescent, Edinburgh EH16 4TJ, UK

**Keywords:** decidua, inflammation, parturition, preterm labour, immune, microarray

## Abstract

**STUDY QUESTION:**

Is labour, both at term and preterm, associated with alterations in decidual lymphocyte densities and widespread changes to the decidual transcriptome?

**SUMMARY ANSWER:**

The onset of parturition, both at term and preterm, is associated with widespread gene expression changes in the decidua, many of which are related to inflammatory signalling, but is not associated with changes in the number of any of the decidual lymphocyte populations examined.

**WHAT IS KNOWN ALREADY:**

Given its location, directly at the maternal–foetal interface, the decidua is likely to play a pivotal role in the onset of parturition, however, the molecular events occurring in the decidua in association with the onset of labour, both at term and preterm, remain relatively poorly defined. Using flow cytometry and microarray analysis, the present study aimed to investigate changes to the immune cell milieu of the decidua in association with the onset of parturition and define the decidual gene signature associated with term and preterm labour (PTL).

**STUDY DESIGN, SIZE, DURATION:**

This study used decidual samples collected from 36 women across four clinical groups: term (38–42 weeks of gestation) not in labour, TNL; term in labour, TL; preterm (<35 weeks of gestation)not in labour, PTNL; and preterm in labour, PTL.

**PARTICIPANTS/MATERIALS, SETTING, METHODS:**

Decidual lymphocytes were isolated from fresh decidual tissue collected from women in each of our four patient groups and stained with a panel of antibodies (CD45, CD3, CD19, CD56, CD4, CD8 and TCRVα24-Jα18) to investigate lymphocyte populations present in the decidua (TNL, *n* = 8; TL, *n* = 7; PTNL, *n* = 5; PTL, *n* = 5). RNA was extracted from decidual tissue and subjected to Illumina HT-12v4.0 BeadChip expression microarrays (TNL, *n* = 11; TL, *n* = 8; PTNL, *n* = 7; PTL, *n* = 10). Quantitative real-time PCR (qRT-PCR) was used to validate the microarray results.

**MAIN RESULTS AND THE ROLE OF CHANCE:**

The relative proportions of decidual lymphocytes (T cells, NK cells, B cells and invariant natural killer (iNKT) cells) were unaffected by either gestation or labour status. However, we found elevated expression of the non-classical MHC-protein, CD1D, in PTL decidua samples (*P* < 0.05), suggesting the potential for increased activation of decidual invariant NKT (iNKT) cells in PTL. Both term and PTL were associated with widespread gene expression changes, particularly related to inflammatory signalling. Up-regulation of candidate genes in TL (*IL-6, PTGS2, ATF3, IER3* and *TNFAIP3*) and PTL (*CXCL8, MARCO, LILRA3* and *PLAU*) were confirmed by qRT-PCR analysis.

**LARGE SCALE DATA:**

Microarray data are available at www.ebi.ac.uk/arrayexpress under accession number E-MTAB-5353.

**LIMITATIONS REASONS FOR CAUTION:**

Whilst no changes in lymphocyte number were observed across our patient samples, we did not investigate the activation state of any of the immune cell sub-populations examined, therefore, it is possible that the function of these cells may be altered in association with labour onset. Additionally, the results of our transcriptomic analyses are descriptive and at this stage, we cannot prove direct causal link with the up-regulation of any of the genes examined and the onset of either term or PTL.

**WIDER IMPLICATIONS OF THE FINDINGS:**

Our findings demonstrate that the onset of parturition is associated with widespread changes to the decidual transcriptome, and there are distinct gene expression changes associated with term and PTL. We confirmed that an inflammatory signature is present within the decidua, and we also report the up-regulation of several genes involved in regulating the inflammatory response. The identification of genes involved in regulating the inflammatory response may provide novel molecular targets for the development of new, more effective therapies for the prevention of preterm birth (PTB). Such targets are urgently required.

**STUDY FUNDING AND COMPETING INTEREST(S):**

This work was supported by Medical Research Council (grant number MR/L002657/1) and Tommy's, the baby charity. Jane Norman has had research grants from the charity Tommy's and from the National Institute for Health Research on PTB during the lifetime of this project. Jane Norman also sits on a data monitoring committee for GSK for a study on PTB prevention and her institution receives financial recompense for this. The other authors do not have any conflicts of interest to declare.

## Introduction

Preterm birth (PTB), defined as birth before 37 weeks of gestation, remains a major public health concern. Rates of PTB have remained relatively unchanged in recent decades, with data from many countries actually demonstrating increased PTB rates (Norman *et al.*, 2009; Blencowe *et al.*, 2012). Globally, PTB is estimated to account for 11.1% of all live births, resulting in ~15 million babies being born prematurely each year (Blencowe *et al.*, 2012). Despite, significant medical advances in the care of premature babies, PTB remains the single biggest cause of neonatal death and the second most common cause of deaths in children under five (Blencowe *et al.*, 2013). Current therapeutic options to stop preterm labour (PTL) are limited and largely ineffective (Norman and Shennan, 2013). This is likely due to the fact that the primary therapy is treatment with a tocolytic agent, but these drugs only target myometrial contractions, which are a single end-point in the cascade of events leading to parturition (Haas *et al.*, 2012). The development of novel, more effective, therapies is hindered by a lack of understanding of the underlying mechanisms leading to parturition, both physiologically at term, and in pathological PTL.

Studies over recent years have conclusively shown that parturition is an inflammatory event associated with an infiltration of immune cells into the cervix, myometrium and foetal membranes with increased production of pro-inflammatory mediators in the utero-placental tissues (Denison *et al.*, 1998; Thomson *et al.*, 1999; Sennstrom *et al.*, 2000; Young *et al.*, 2002; Osman *et al.*, 2003). These findings have been supported by genome-wide analyses of gestational tissues, which have demonstrated that labour at term is associated with a core inflammatory response (Hassan *et al.*, 2006; Bollapragada *et al.*, 2009; Mittal *et al.*, 2010; Stephen *et al.*, 2015; Sharp *et al.*, 2016). PTL is thought to occur via the premature activation of similar inflammatory events, but importantly the underlying mechanism activating these inflammatory cascades are likely to differ between term and PTL, with PTL thought to occur in response to pathological processes, such as intrauterine infection, decidual haemorrhage or stress (Rinaldi *et al.*, 2011; Romero *et al.*, 2014).

The majority of research has focussed on defining the molecular events underlying myometrial contractions, foetal membrane rupture and cervical ripening, with less focus being given to the role of the decidua in the onset of labour. Given that the uterine decidua is located directly at the maternal–foetal interface, it is ideally placed to have a key role in the events leading to parturition. During pregnancy, the decidua can be subdivided depending on the point of contact with foetal tissues. The decidua basalis, describes the decidual tissue which surrounds the placenta and invading interstitial trophoblasts, while the decidua parietalis, describes the decidual tissue which is in contact with the foetal membranes, specifically the chorion layer (Cunningham *et al.*, 2014).

Studies examining the changes in decidual immune cell populations with the onset of labour have provided conflicting results, with one study reporting no changes in leucocyte population (Osman *et al.*, 2003), while others have reported increases in NK cells, T cells and macrophages, in term labour (Sindram-Trujillo *et al.*, 2004; Hamilton *et al.*, 2012; Gomez-Lopez *et al.*, 2013). The decidual leucocyte changes occurring in PTL have been less well examined, but elevated macrophage, neutrophil, T cell and NK cell numbers in the decidua have been reported in women with PTL (Hamilton *et al.*, 2012). Rodent studies have demonstrated that this decidual leucocyte infiltration precedes the onset of parturition (Hamilton *et al.*, 2012; Shynlova *et al.*, 2013b; Rinaldi *et al.*, 2014), suggesting that alterations in the decidual immune cell populations may be an important early event in the inflammatory events surrounding labour.

There has been a long-standing interest in defining the role of the decidua in the onset of parturition, with ‘decidual activation’ being proposed as critical to labour in the 1980s (Casey and MacDonald, 1988). Elevated prostaglandin output and the production of pro-inflammatory mediators, such as cytokines and matrix metalloproteinases (MMPs), from the decidua is thought to stimulate myometrial contractions and extracellular matrix (ECM) remodelling in the foetal membranes and cervix, thus stimulating the progression of labour (Makino *et al.*, 2007; Snegovskikh *et al.*, 2011). A recent review by Norwitz *et al.* (2015), proposes that the decidua is key to triggering the cascade of events, which result in the onset of labour. During pregnancy, the decidua is integral to maintaining uterine quiescence; with advancing gestational age, there is withdrawal of active suppression and/or the enhanced ability of the decidua to induce inflammatory signals, which results in the initiation of the pro-inflammatory signalling cascade, culminating in parturition. In this theory, PTB is proposed to occur as a result of premature dysregulation of decidual inflammation, which could be induced by the pathologic processes already mentioned (Norwitz *et al.*, 2015).

Hence, it is clear that improving our understanding of the molecular mechanisms taking place in the decidua during labour, both at term and preterm, is critical to the development of novel therapies to delay PTL and reduce the incidence of PTB. In this study we aimed to investigate changes in the decidual lymphocyte populations in relation to labour onset, both at term and preterm, and to use microarray analysis to determine the decidual gene signature associated with term and preterm parturition. We hypothesized that the onset of labour, both at term and preterm, will be associated with changes to the decidual lymphocyte populations, that labour will result in widespread changes to the decidual transcriptome relating to inflammatory gene signalling and these changes may differ in the context of physiological term labour and pathological PTL.

## Materials and Methods

### Sample collection

Decidual tissue was isolated from foetal membrane samples collected from women who delivered at Edinburgh Royal Infirmary, with written and informed consent, according to the ethical approval and governance granted to the Edinburgh Reproductive Tissues BioBank by the West of Scotland Research Ethics Committee 4 (REC reference: 09/S0704/3) until 30/09/2014; and consequently by the East of Scotland Research Ethics Service Tayside Committee on Medical Research Ethics B (REC reference: 13/ES/0126).

Samples were collected from women delivering at term (38–42 weeks of gestation) and preterm (<35 weeks of gestation). A total of 36 women were recruited from four patient groups: term not in labour (TNL; *n* = 11), term in labour (TL; *n* = 8), preterm not in labour (PTNL; *n* = 7) and preterm in labour (*n* = 10). TNL and PTNL samples were collected from women following caesarean section delivery; TL and PTL samples were collected following either vaginal delivery or emergency caesarean section delivery. Patient characteristics are detailed in Table I.

**Table I:** Demographic and clinical characteristics of the study groups^a^.

	TNL (*n* = 11)	TL (*n* = 8)	PTNL (*n* = 7)	PTL (*n* = 10)
Maternal age (year)	34 (30–43)	26 (22–40)	34 (18–39)	30 (16–39)
Parity^b^	1 (0–2)	0 (0–1)	0 (0–2)	0 (0–3)
BMI (kg/m^2^)^c^	22.7 (19.9–28.1)	27.1 (21.0–28.7)	24.4 (22.1–26.2)	24.8 (19.5–30.0)
Gestation at delivery (week)	39.3 (39.0–41.0)	40.1 (38.2–41.1)	32.4 (29.0–34.4)*^##^	30.4 (24.0–34.6)**^####^
Indication for caesarean section (*n*)				
Foetal distress	0	2	0	3
Failure to progress	0	3	0	0
Breech presentation	2	0	0	2
Obstetric history	6	0	0	0
IUGR	0	0	4	0
Pre-eclampsia	0	0	2	0
Other or missing	3	3	1	5
Foetal membrane rupture				
Spontaneous	0	5	0	2
Artificial	0	3	0	2
PPROM	0	0	0	6
None or missing	11	0	7	0
Evidence of chorioamnionitis: *n* (%)^d^			0 (0%)	5 (50%)
Evidence of placental abruption: *n* (%)^e^			0 (0%)	1 (10%)

^a^The numbers are median and range unless otherwise indicated.

^b^Parity data were missing from one patient in the PTL group.

^c^BMI at booking to antenatal care. Due to patient transfer, BMI was not available for three PTNL patients and three PTL patients.

^d^Chorioamnionitis status from available pathology reports for preterm patients. Pathology reports were not available for two PTL patients.

^e^Placental abruption confirmed from available pathology reports from preterm patients. Pathology reports were not available for two PTL patients.

*P < 0.05, **P < 0.01 versus TNL; ^##^*P* < 0.01, ^####^*P* < 0.0001 versus TL; Kruskal–Wallis with *post hoc* Dunn test.

Following delivery, full-thickness foetal membranes were cut from the placenta and placed in PBS. Samples were either stored at 4°C for no more than 1–5 h before processing, or processed immediately following collection. Amnion was removed from the foetal membranes and decidua parietalis tissue was carefully scraped from the chorion. Samples of decidual tissue were either frozen at −80°C for further RNA and protein analysis or digested for isolation of decidual lymphocytes.

### Isolation of decidual lymphocyte populations

Decidual tissue was finely minced with scissors and digested overnight at room temperature with agitation in 20 ml of RPMI 1640 (Gibco, Life Technologies Ltd, Paisley, UK) containing 20% FCS (Gibco), 0.125 mg/ml collagenase type IV (Sigma-Aldrich, Poole, UK) and 0.1 mg/ml DNase I (Sigma). The digested tissue was then sequentially filtered through 70 μm and 40 μm cell strainers (BD Biosciences, Oxford, UK) to obtain a single cell suspension and centrifuged at 800 *g* for 5 min. The cell pellet was resuspended in 3 ml PBS (Gibco) + 2% FCS and the decidual lymphocytes were isolated by density gradient centrifugation using Histopaque 1077 (Sigma). The isolated immune cells were washed in 20 ml PBS centrifuged at 800 *g* for 15 min and resuspended in 1 ml PBS for cell counting and flow cytometry analysis.

### Flow cytometry

Flow cytometry was used to investigate the decidual lymphocyte populations present in term and preterm in labour and non-labouring samples. Due to the small amount of foetal membrane tissue isolated from preterm patients, it was not always possible to isolate a large enough population of decidual mononuclear cells for flow cytometry analysis. Therefore sample sizes for the flow cytometry analysis were: TNL (*n* = 8), TL (*n* = 7), PTNL (*n* = 5) and PTL (*n* = 5). Isolated lymphocytes were incubated with the following panel of antibodies for 30 min on ice: CD45-FITC (1:5, BioLegend, Cambridge, UK), CD3-PerCP/Cy5.5 (1:100, BioLegend), TCRVα24-Jα18-PE (1:50; BioLegend), CD56-BV421 (1:100; BioLegend), CD4-BV570 (1:20; BioLegend), CD8-APC/Cy7 (1:50; BioLegend) and CD19-PE/Cy7 (1:50; BioLegend). Following this, samples were centrifuged for 5 min at 350 *g*, the supernatant was removed and the samples resuspended in 200 μl PBS + 1% FCS for flow cytometric analysis. The nuclear stain DAPI was added to the samples as a live/dead marker. Analysis was carried out using the BD LSR Fortessa and data were collected using BD FACSDiva software and analyised using FlowJo software (Treestar, Ashland, OR, USA).

### RNA extraction and sample preparation

Total RNA was extracted from decidua samples using TRI Reagent and RNeasy mini kit (Qiagen, Crawley, UK) as per the manufacturer's guidelines. The quantity and quality of RNA was assessed using a Nano-Drop ND 1000 spectrophotometer (Thermo Scientific, UK). In preparation for the microarray experiment, 750ng total RNA was amplified and biotin-labelled using the Illumina TotalPrep RNA Amplification Kit (Ambion, UK). The quantity and quality of the biotin-labelled cRNA samples was further assessed by the Wellcome Trust Clinical Research Facility, Edinburgh using a Bioanalyzer 2100, according to their protocols (Agilent Technologies LDA UK Limited, Cheshire, UK).

### Illumina HT-12v4.0 beadchip expression microarray

Samples were randomly split over three Illumina HT-12 v4.0 BeadChips to minimize the effects of inter-chip variability. The chips were imaged using a BeadArray Reader and raw data were obtained with Illumina BeadStudio software. Raw data are available at www.ebi.ac.uk/arrayexpress under accession number E-MTAB-5353.

### Microarray analysis

Microarray data analysis was performed by Fios Genomics Ltd (Bioquarter, Edinburgh, UK). The analysis included quality control and exploratory analysis of the data sets followed by identification of differentially expressed genes (DEGs) and also functional analysis for enrichment of KEGG pathways amongst the DEGs.

Raw data underwent quality control analysis using the arrayQualityMetrics package in Bioconductor (Kauffmann and Huber, 2010) to identify outliers. Two samples (both from PTL group) failed the quality control checks and were removed from further statistical analysis of the microarray data. The arrayQualityMetrics analysis scored the samples on the basis of four metrics (maplot, boxplot, heatmap and manual inspection). Boxplots of the raw, transformed and normalized data were also assessed manually, as were outlier and sample relation plots were generated for all stages of the processing. One PTL sample failed the automatic quality checks based on two metrics (Boxplot and Heatmap) and was removed from downstream analyses. The second PTL sample passed the automatic quality checks as it only failed one metric (Heatmap). However after manual inspection it clearly appeared to be an outlier (PCA plot, dendrogram and boxplot) and was therefore removed from downstream analyses. Data were normalized using robust spline normalization after being subjected to a variance stabilizing transformation.

### Network graph analysis

To further analyse the microarray expression data and examine whether samples within the four patient groups had similar expression profiles, we created a sample–sample network graph in BioLayout *Express*^3D^, as described (Theocharidis *et al.*, 2009; Sharp *et al.*, 2016). The normalized expression data for the top up-and down-regulated genes (*P* < 0.05, fold change ≥1.2) were used for this analysis. In the sample–sample network graph, each ‘node’ represents a sample which is connected to other samples by ‘edges’ weighted according to the strength of the sample–sample Pearson's correlation coefficient. All correlation values above 0.85 were used to draw a graph of this similarity matrix. In this network graph, if samples have similar gene expression signatures they appear closer to each other in the graph, thus creating a local structure. The Markov Clustering algorithm (MCL) (Enright *et al.*, 2002) was performed with the inflation value (MCLi) set at 20.0 to give an unbiased assessment of how the samples cluster. This can then be further examined by colouring the nodes according to gestation and labour status to assess whether different patient groups have similar gene expression profiles.

### Quantitative real-time PCR

To validate the results of the microarray experiment, total RNA (300ng) was reverse transcribed using the High Capacity cDNA Reverse Transcription kit (Applied Biosystems). QRT-PCR was carried out to quantify the mRNA expression of specific genes of interest. Details of the pre-designed gene expression assays from Applied Biosystems used are given in Supplementary Table I. Target gene expression was normalized for RNA loading using *ACTB* and the expression in each sample was calculated relative to the mean of the TNL samples, using the 2^−ΔΔCt^ method of analysis. All qRT-PCR analysis was performed on an Applied Biosystems 7900HT instrument.

### Western blotting

Total decidual protein was extracted in RIPA buffer (Sigma) supplemented with cOmplete™ Protease Inhibitor Cocktail tablet (Sigma). Briefly, 20 μg of extracted protein was separated on NuPAGE 4–12% Bis-Tris precast gels (Invitrogen, Life Technologies Ltd, Paisley, UK) and transferred to Immobilon-FL polyvinylidene difluoridemembrane (Millipore, Hertfordshire, UK). Membranes were blocked in 5% Milk/TBS/0.001% Tween-20 for 1 h at room temperature and incubated overnight with at 4°C with the primary antibodies: mouse anti-CD1d (1:250; MAB6979, R&D Systems, Abingdon, UK) and rabbit anti-β-actin (1:2500; ab8227, Abcam, Cambridge, UK). To detect the bound proteins, the membrane was incubated with two fluorescently-labelled secondary antibodies, IRDye 800CW and IRDye 680RD (1:10 000; Li-Cor Biosciences, Nebraska, USA), to detect both the protein of interest and the β-actin loading control protein simultaneously. Bands were visualized using a Li-Cor Odyssey Infrared Imaging System and analysed using Image Studio Software (Li-Cor Biosciences). The intensity of CD1d fluorescence was calculated relative to β-actin.

### Statistics

Data are presented as mean ± SEM. Data were analysed for normal distribution using the Shapiro–Wilk normality test. The specific statistical analysis tests performed for the different data sets are detailed in the figure legends. Statistical analyses for the decidual immune cell populations, qRT-PCR and western blotting were performed using GraphPad Prism 6.0 software (GraphPad, San Diego, CA, USA). *P* < 0.05 was considered statistically significant.

## Results

### Decidual lymphocyte sub-populations in term and preterm labouring and non-labouring samples were not significantly different

**Figure 1:**
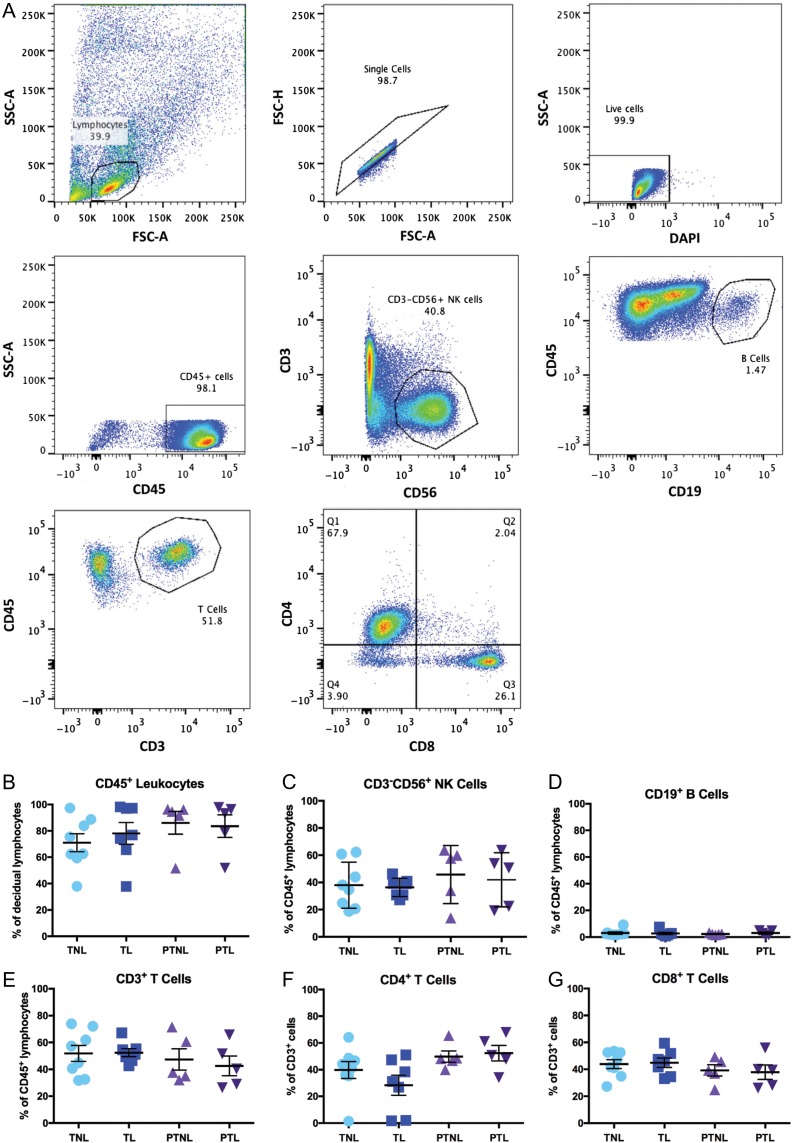
Decidual immune cell population analysis in term and PTL. Flow cytometric analysis of isolated decidual mononuclear cells from four patient groups: TNL (*n* = 8), TL (*n* = 7), PTNL (*n* = 5) and PTL (*n* = 5). Cells were stained with antibodies raised against the following antigens: CD45, CD3, CD56, CD19, CD4 and CD8. (**A**) Gating strategy. Lymphocytes were gated based on their forward light scatter (FSC) and side light scatter (SSC), doublet cells were removed and live cells were selected based on exclusion of DAPI. Individual cell populations were then identified based on antibody staining. (**B**) Proportion of CD45^+^ lymphocytes out of all live cells within lymphocyte gate. (**C**) Proportion of CD3−CD56^+^ NK cells within the CD45^+^ lymphocyte gate. (**D**) Proportion of CD19^+^ B cells within the CD45^+^ lymphocyte gate. (**E**) Proportion of CD3^+^ T cells within the CD45^+^ lymphocyte gate. (**F**) Proportion of CD4^+^ T cells within the CD3^+^ T cell gate. (**G**) Proportion of CD8^+^ T cells within the CD3^+^ T cell gate. Data presented as mean ± SEM. Data were analysed by Kruskal–Wallis but no significant differences were found. PTL, preterm labour.

Flow cytometry analysis was carried out to characterize the decidual lymphocyte populations present both at term and preterm, in labouring and non-labouring samples. The gating strategy used is summarized in Fig. 1A. The percentage of decidual CD45^+^ cells (expressed as a proportion of all decidual lymphocytes) was found to be similar at both term and preterm and in labouring and non-labouring samples (Fig. 1B). The majority of CD45^+^ lymphocytes were made up of NK cells (Fig. 1C) and T cells (Fig. 1E), with B cells (Fig. 1D) accounting for a much smaller proportion of the decidual CD45^+^ lymphocyte population. The analysis did not reveal any statistically significant differences in the proportion of any of these immune cell subsets when comparing TNL, TL, PTNL and PTL groups. Further analysis of the CD3^+^ T cells revealed that both CD4^+^ (Fig. 1F) and CD8^+^ (Fig. 1G) T cells are present within the decidua, and we did not find any effect of gestation or labour status on the proportion of these cell types.

### Decidual iNKT cells did not differ in term and PTL but their activation status may change

**Figure 2:**
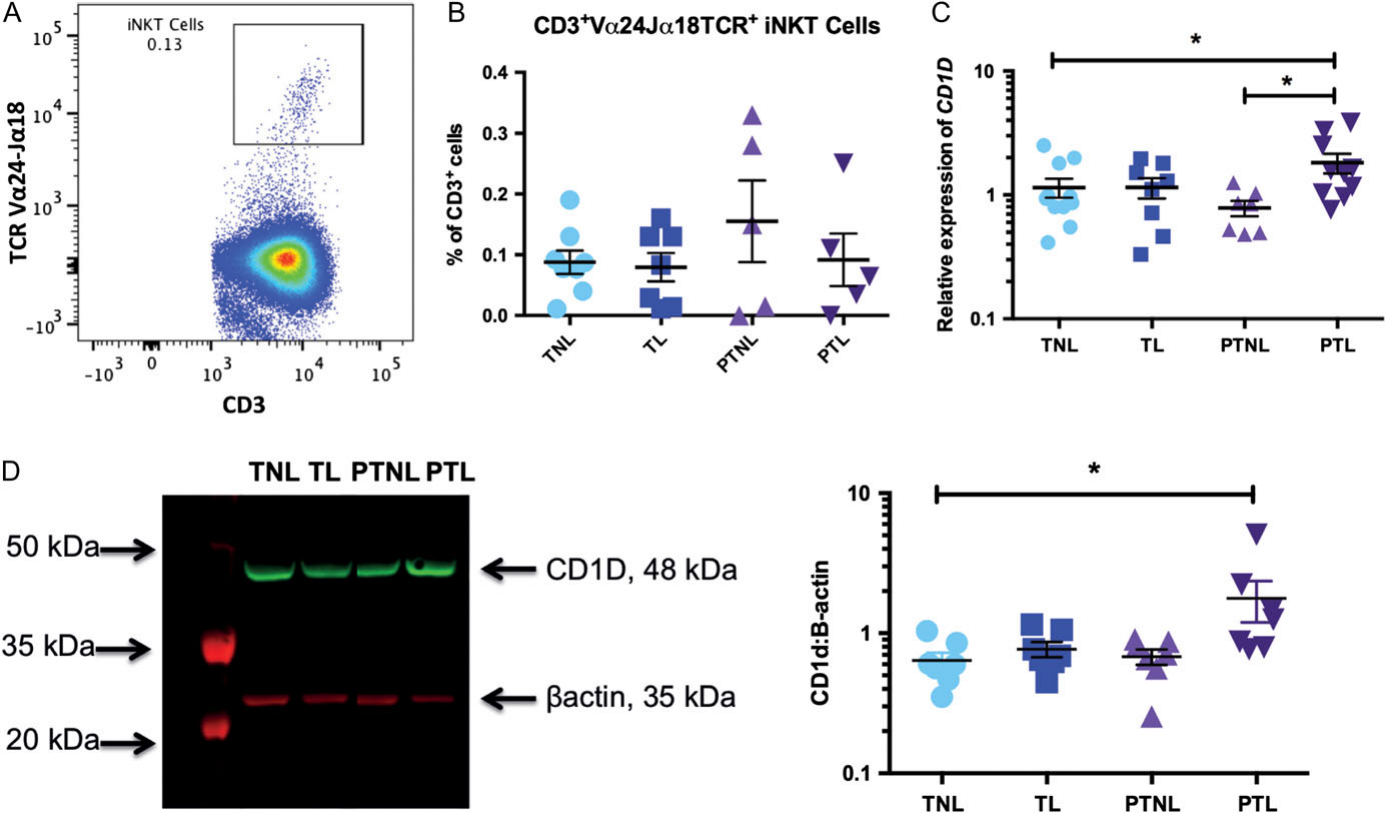
Decidual iNKT cells and CD1d in term and PTL. The presence of a decidual iNKT cell population was examined using flow cytometry in samples from our four patient groups: TNL (*n* = 8), TL (*n* = 7), PTNL (*n* = 5) and PTL (*n* = 5). (**A**) Decidual iNKT cells were gated based on their expression of CD3 and TCRVα24 Jα18. (**B**) Proportion of iNKT cells within the CD3^+^ gate. Decidual expression of CD1d was examined at the mRNA and protein level by qRT-PCR and western blotting, respectively. (**C**) Decidual *CD1D* mRNA expression [TNL (*n* = 11); TL (*n* = 8), PTNL (*n* = 7), PTL (*n* = 10)]. (**D**) Decidual CD1d protein expression (*n* = 7 in each group). Data presented as mean ± SEM. QRT-PCR data analysed by one-way ANOVA followed by Newman–Keuls *post hoc* test; flow cytometry and western blot data analysed by Kruskal–Wallis followed by Dunn *post hoc* test. *P < 0.05. iNKT, invariant natural killer.

Given the recent studies suggesting that decidual invariant natural killer (iNKT) cells may play a pathological role in the onset of PTL (Li *et al.*, 2012, 2015; St Louis *et al.*, 2016), we also examined whether these cells were present in our samples. We found a very small population of CD3^+^ TCRVα24-Jα18^+^present in our decidual samples (TNL: 0.09% ± 0.02, TL: 0.08% ± 0.02, PTNL: 0.16% ± 0.07, PTL: 0.09 ± 0.04 of decidual CD3^+^ cell population; Fig. 2A), but as with the other immune cell subsets examined, we did not find a statistically significant difference in the number of iNKT cells when we compared TNL, TL, PTNL and PTL samples (Fig. 2B). However, we did find that the expression *CD1D*, which encodes for the non-classical MHC-protein CD1d and is involved in iNKT cell activation, is significantly elevated in decidual samples collected from PTL women, compared to both PTNL and TNL (2.3-fold and 1.6-fold greater, respectively; *P* < 0.05; PTL mean relative expression, 1.82 ± 0.33; TNL mean relative expression, 1.15 ± 0.20; PTNL mean relative expression, 0.78 ± 0.11; Fig. 2C). Decidual PTL samples also had significantly elevated CD1d protein levels compared with TNL samples (2.8-fold greater; *P* < 0.05; PTL mean relative expression: 1.78 ± 0.58; TNL mean relative expression: 0.64 ± 0.09; Fig. 2D).

### Microarray analysis of the decidual transcriptome in term and preterm samples revealed distinct but overlapping gene signatures

To explore the gene signatures activated in the decidua during labour, both physiologically at term and pathologically in PTL, we performed whole genome microarray analyses on decidual samples collected from women in each of our four patient groups, TNL, TL, PTNL and PTL. DEGs were identified as those with a fold change of ≥1.2 and *P* < 0.01. Comparisons between the different patient groups identified 104 DEGs between TL and TNL samples (56 up- and 48 down-regulated in TL); 112 DEGs between TL and PTL samples (32 up- and 80-down-regulated in PTL); and 129 DEGs between PTL and PTNL samples (96 up- and 33-down-regulated in PTL). The top 10 DEGs in each comparison (TL vs TNL; TL vs PTL and PTL vs PTNL) are shown in Table II. The full list of DEGs for each comparison is shown in Supplementary Table II.

**Table II:** Top 10 DEGs.

Comparison	Symbol	Description	Fold change	*P*-value
TL vs TNL	*TNFAIP3*	tumour necrosis factor, alpha-induced protein 3	2.716	1.40E–04
	*IER3*	immediate early response 3	2.68	2.37E–03
	*IL-6*	interleukin 6	2.657	8.79E–03
	*PTGS2*	prostaglandin-endoperoxide synthase 2	2.474	2.15E–03
	*ATF3*	activating transcription factor 3	2.31	6.30E–03
	*PHLDA1*	pleckstrin homology-like domain, family A, member 1	2.147	3.98E–03
	*DUSP5*	dual specificity phosphatase 5	2.066	3.64E–03
	*NFKBIA*	nuclear factor of kappa light polypeptide gene enhancer in B-cells inhibitor, alpha	1.988	8.04E–04
	*ZC3H12A*	zinc finger CCCH-type containing 12 A	1.905	3.84E–03
	*DDIT4*	DNA-damage-inducible transcript 4	1.824	4.79E–03
	*MMP7*	matrix metallopeptidase 7 (matrilysin, uterine)	−2.387	1.84E–03
	*ZC3HAV1*	zinc finger CCCH-type, antiviral 1	−1.564	1.51E–05
	*COL6A3*	collagen, type VI, alpha 3	−1.538	4.49E–03
	*CIRBP*	cold inducible RNA binding protein	−1.456	1.88E–03
	*NID1*	nidogen 1	−1.396	7.91E–03
	*CYB561D1*	cytochrome b561 family, member D1	−1.394	2.64E–03
	*CLSTN1*	calsyntenin 1	−1.355	9.51E–03
	*LHPP*	phospholysine phosphohistidine inorganic pyrophosphate phosphatase	−1.338	2.94E–03
	*RAB22A*	RAB22A, member RAS oncogene family	−1.327	9.02E–04
	*CCDC106*	coiled-coil domain containing 106	−1.303	6.30E–03
TL vs PTL	*FOSB*	FBJ murine osteosarcoma viral oncogene homologue B	2.538	9.45E–03
	*FST*	follistatin	2.325	3.96E–03
	*HES4*	hes family bHLH transcription factor 4	1.691	9.09E–04
	*KLF4*	Kruppel-like factor 4 (gut)	1.563	9.62E–03
	*SASH1*	SAM and SH3 domain containing 1	1.493	8.10E–03
	*HRK*	harakiri, BCL2 interacting protein	1.492	9.84E–03
	*NEDD9*	neural precursor cell expressed, developmentally down-regulated 9	1.489	5.24E–03
	*ELMSAN1*	ELM2 and Myb/SANT-like domain containing 1	1.44	2.50E–03
	*FOXC1*	forkhead box C1	1.418	4.11E–03
	*CCNB1IP1*	cyclin B1 interacting protein 1, E3 ubiquitin protein ligase	1.414	6.07E–03
	*FGA*	fibrinogen alpha chain	−4.108	6.28E–03
	*FGG*	fibrinogen gamma chain	−3.688	5.88E–04
	*FGB*	fibrinogen beta chain	−2.753	2.53E–03
	*PLAT*	plasminogen activator, tissue	−2.235	5.73E–03
	*MARCO*	macrophage receptor with collagenous structure	−2.087	4.03E–03
	*LILRA3*	leucocyte immunoglobulin-like receptor, subfamily A (without TM domain), member 3	−1.945	9.45E–03
	*NR1H3*	nuclear receptor subfamily 1, group H, member 3	−1.769	8.67E–03
	*KCNK4*	potassium channel, subfamily K, member 4	−1.75	1.95E–03
	*TNFRSF8*	tumour necrosis factor receptor superfamily, member 8	−1.72	1.53E–03
	*WARS*	tryptophanyl-tRNA synthetase	−1.712	9.12E–03
PTL vs PTNL	*CXCL8*	chemokine (C-X-C motif) ligand 8	5.441	2.49E–03
	*CEMIP*	cell migration inducing protein, hyaluronan binding	3.338	6.97E–03
	*S100A8*	S100 calcium binding protein A8	3.193	1.02E–03
	*FGG*	fibrinogen gamma chain	3.023	3.76E–03
	*SOD2*	superoxide dismutase 2, mitochondrial	2.89	2.04E–03
	*FGB*	fibrinogen beta chain	2.784	3.08E–03
	*NAMPT*	nicotinamide phosphoribosyltransferase	2.742	1.03E–03
	*FGG*	fibrinogen gamma chain	2.687	9.45E–04
	*S100A9*	S100 calcium binding protein A9	2.547	9.93E–04
	*AQP9*	aquaporin 9	2.528	3.60E–03
	*FOSB*	FBJ murine osteosarcoma viral oncogene homologue B	−2.652	8.73E–03
	*TACSTD2*	tumour-associated calcium signal transducer 2	−2.334	9.47E–03
	*MGAT3*	mannosyl (beta-1,4-)-glycoprotein beta-1,4-N-acetylglucosaminyltransferase	−1.702	2.04E–03
	*SASH1*	SAM and SH3 domain containing 1	−1.536	6.36E–03
	*CYB561D1*	cytochrome b561 family, member D1	−1.467	1.96E–03
	*FAM53B*	family with sequence similarity 53, member B	−1.448	5.53E–03
	*CRYZ*	crystallin, zeta (quinone reductase)	−1.438	9.11E–03
	*C22orf29*	chromosome 22 open reading frame 29	−1.436	2.44E–03
	*ZHX3*	zinc fingers and homeoboxes 3	−1.428	2.39E–04
	*VTCN1*	V-set domain containing T cell activation inhibitor 1	−1.412	1.12E–03

DEG, differentially expressed gene.

**Figure 3:**
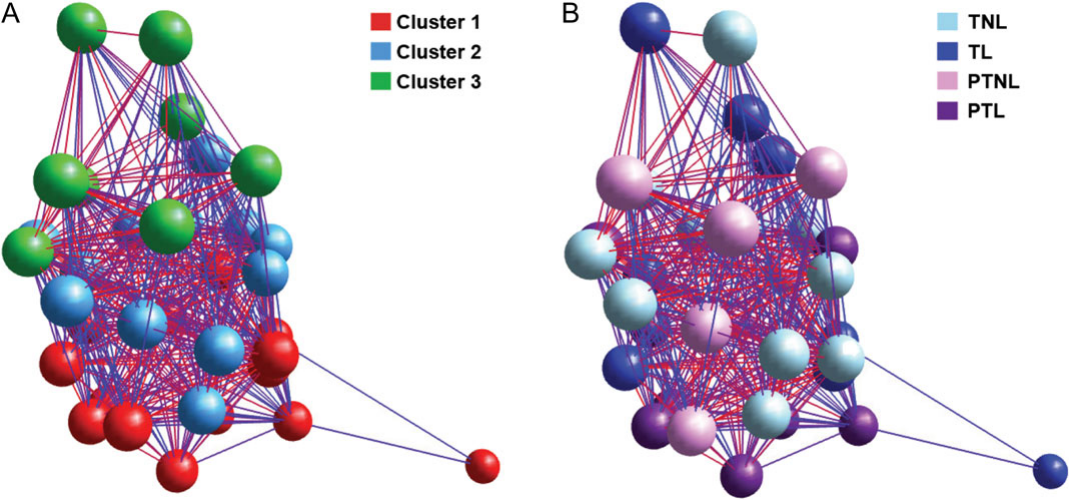
Sample–sample network graph of microarray data. Normalized expression data for the top up-and down-regulated genes (*P* < 0.05, fold change ≥ 1.2) identified by microarray analysis were visualized using BioLayout *Express*^3D^. Each node represents a different patient sample and edges are coloured to reflect the Pearson correlation that they represent. Red edges indicate high correlation, and blue edges represent low correlation. The same data set is used for each graph. (**A**) unbiased MCL cluster (MCLi = 20). (**B**) Nodes are coloured by gestation and labour status into our four sample groups: TNL (*n* = 11), TL (*n* = 9), PTNL (*n* = 7) and PTL (*n* = 8).

To determine whether there was any sample–sample correlation in the gene signatures identified by the microarray, we also used a network graph approach to analyse the microarray data. The sample–sample network graph was generated using the normalized expression data for the top up- and down-regulated genes (*P* < 0.05, fold change ≥ 1.2). Using MCL clustering (MCLi = 20), three separate clusters were identified (Fig. 3A). To determine whether there was any relationship between the expression data and gestation and/or labour status, the nodes were coloured according to patient group (Fig. 3B). This analysis revealed that samples from each patient group had quite diverse gene expression signatures, as not all samples from any patient group belonged to a single cluster. However, cluster one is predominantly made up of labouring samples (10 out of the 14 samples in cluster one come from labouring groups, with six of these samples belonging to the PTL group and four belonging to the TL group), suggesting that there are similarities in gene expression in the labouring samples. Conversely, cluster two is predominantly made up of nodes belonging to TNL patients (7 out of 11 TNL samples; 2 out of 8 TL samples; 2 out of 8 PTL samples; and 1 out of 7 PTNL samples). Cluster three contains the remaining samples (3 out of 11 TNL samples; 2 out of 8 TL samples; and 3 out of 7 PTNL samples). Therefore, this network graph approach reveals that there is quite high variation in decidual gene expression within the four patient groups. Importantly, however, 75% of PTL samples are contained within cluster 1, suggesting that there may be a distinct decidual gene expression signature associated with PTL.

### Decidual gene signature associated with term labour

To further investigate the gene signature of the decidua during physiological labour at term, the significantly DEGs in the TL versus TNL comparison were assessed for KEGG pathway enrichment. This analysis identified several enriched KEGG pathways in up-regulated features and one enriched KEGG pathway in down-regulated features at *P* < 0.05 in TL decidua, compared with TNL decidua (Supplementary Table III). The significantly enriched KEGG pathways in TL samples included a number of pathways involved in inflammation and immunity (e.g. ‘TNF-signalling pathway’, ‘NOD-like receptor signalling’ and ‘NF-kappa B signalling’).

**Figure 4:**
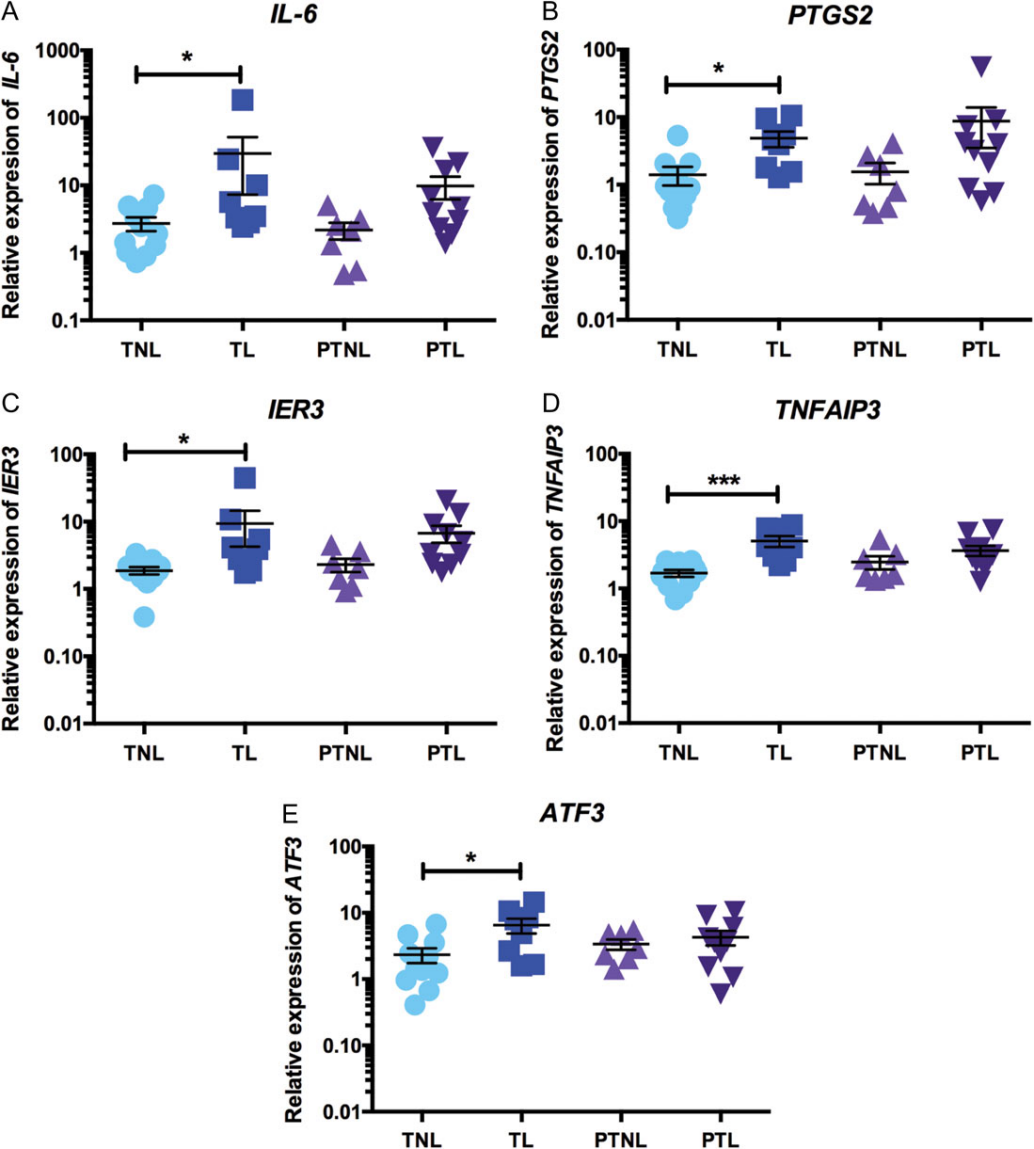
QRT-PCR validations of gene expression changes in TL decidua samples. Decidual expression of selected genes identified as significantly elevated in TL in the microarray analysis was examined across our four sample groups by qRT-PCR [TNL (*n* = 11), TL (*n* = 9), PTNL (*n* = 7) and PTL (*n* = 10)]. (**A**) *IL-6* expression, (**B**) *PTGS2* expression, (**C**) *IER3* expression, (**D**) *TNFAIP3* expression and (**E**) *ATF3* expression. Data are presented as mean fold change ± SEM. Data were analysed by one-way ANOVA followed by Tukey *post hoc* test. *P < 0.05, ***P < 0.001.

We identified a number of genes of interest that were found to be significantly up-regulated in TL versus TNL samples in the microarray and examined whether the microarray results could be validated by qRT-PCR. Our qRT-PCR analysis confirmed that several important genes involved in inflammatory signalling are significantly elevated in decidual TL samples, compared with TNL samples. Specifically, we found that *IL-6* expression was 10.9-fold higher in TL samples compared to TNL (*P* < 0.05; TL mean relative expression: 29.54 ± 22.28; TNL mean relative expression: 2.72 ± 0.65; Fig. 4A); expression of *PTGS2* was 3.5-fold higher in TL decidua versus TNL decidua (*P* < 0.05; TL mean relative expression: 4.86 ± 1.30; TNL mean relative expression 1.41 ± 0.43; Fig. 4B); and *IER3* mRNA levels were also 5-fold higher in TL samples, compared with TNL samples (*P* < 0.05; TL mean relative expression: 9.38 ± 5.12; TNL mean relative expression: 1.87 ± 0.23; Fig. 4C). Although the expression of each of these three genes was also found to be elevated in PTL samples, this increase did not reach statistical significance. Interestingly, the expression of the other two genes we examined were only elevated in TL: decidual *TNFAIP3* expression was 3-fold higher in TL samples, compared with TNL (*P* < 0.001; TL mean relative expression: 5.06 ± 0.94; TNL mean relative expression: 1.69 ± 0.20; Fig. 4D); and *ATF3* expression was 2.8-fold higher in TL samples, compared with TNL (*P* < 0.05; TL mean relative expression: 6.54 ± 1.65; TNL mean relative expression: 2.33 ± 0.58; Fig. 4E).

### Decidual gene signature associated with PTL

To examine whether we could identify a specific gene signature associated with PTL in the decidua, the significantly up- and down-regulated genes identified by the microarray were also assessed for KEGG pathway enrichment. This analysis was carried out for two separate comparisons, PTL versus TL and PTL versus PTNL, to identify any specific differences in the labour process between preterm and term labour and to identify gene expression differences between the two pathological conditions of PTL and PTNL. When comparing PTL and TL decidual samples, KEGG pathway analysis identified five enriched pathways in up-regulated features, and three enriched pathways in down-regulated features at *P* < 0.05 (Supplementary Table IV). In this comparison, the top up-regulated pathway in PTL decidua was found to be ‘complement and coagulation cascades’, while ‘mRNA surveillance’ was the top down-regulated pathway. When examining PTL versus PTNL samples, the KEGG pathway enrichment analysis identified eight enriched pathways in up-regulated features, and twelve enriched pathways in down-regulated features at *P* < 0.05 (Supplementary Table V). Similar to the previous comparison, ‘complement and coagulation cascades’ was one of the top up-regulated pathways in decidua from PTL samples, along with ‘cytokine–cytokine receptor interaction’.

**Figure 5:**
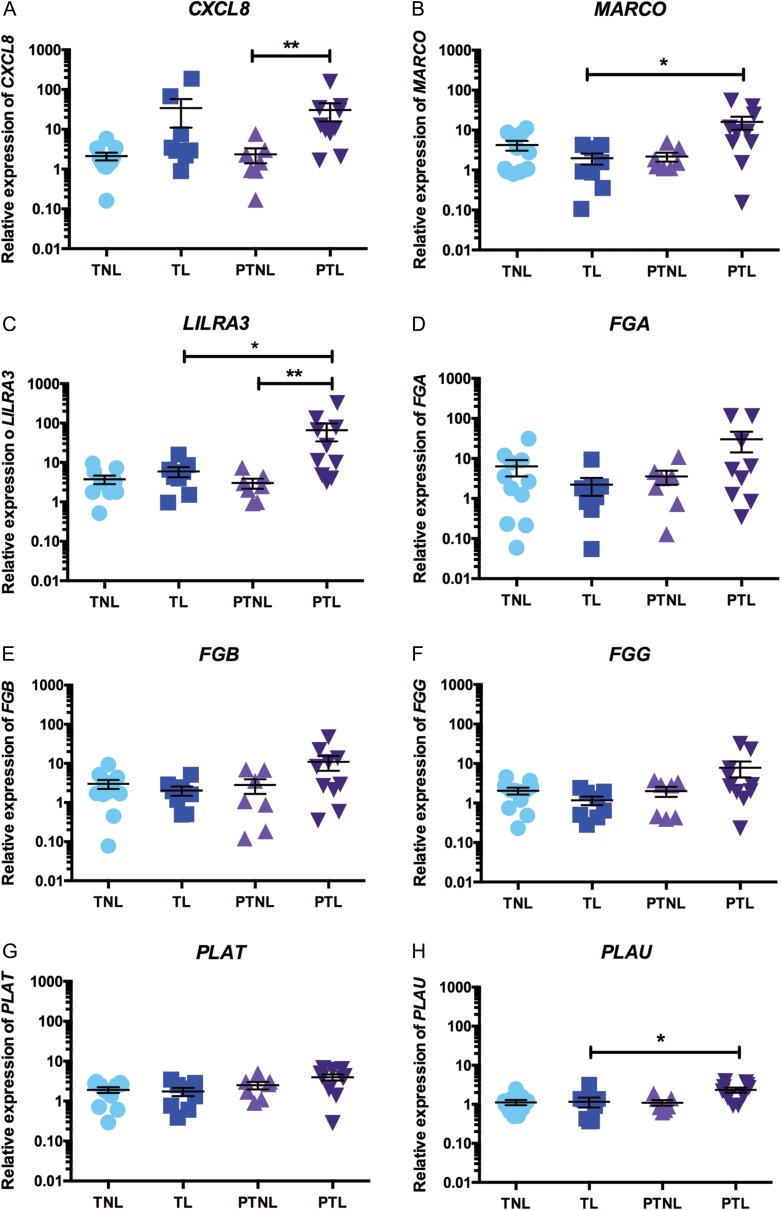
QRT-PCR validations of gene expression changes in PTL decidua samples. Decidual gene expression of selected genes identified as significantly elevated in PTL samples in the microarray analysis was examined across our four sample groups by qRT-PCR [TNL (*n* = 11), TL (*n* = 9), PTNL (*n* = 7), PTL (*n* = 10)]. (**A**) *CXCL8* expression, (**B**) *MARCO* expression, (**C**) *LILRA3* expression, (**D**) *FGA* expression, (**E**) *FGB* expression, (**F**) *FGG* expression, (**G**) *PLAT* expression and (**H**) *PLAU* expression. Data are presented as mean fold change ± SEM. Data were analysed by one-way ANOVA followed by Tukey *post hoc* test. *P < 0.05, **P < 0.01.

To further investigate the genetic signature of the decidua during PTL, we identified several genes of interest that were suggested to be important in PTL from both the DEG lists generated from the microarray data and also from the KEGG pathway enrichment analysis, and aimed to validate these genes by qRT-PCR analysis. In accordance with the microarray results, we found that expression of *CXCL8* was 13-fold greater in decidua samples collected from women in PTL, compared to PTNL samples (*P* < 0.01; PTL mean relative expression: 30.5 ± 14.8; PTNL mean relative expression: 2.37 ± 0.96; Fig. 5A). Similarly, decidual expression of *MARCO* and *LILRA3* was also significantly elevated in PTL samples (*MARCO:* 8.1-fold, *P* < 0.05 vs TL; PTL mean relative expression: 16.1 ± 5.7; TL mean relative expression: 1.98 ± 0.62; Fig. 5B; *LILRA3:* 11-fold, *P* < 0.05 vs TL; 21.8-fold, *P* < 0.01 vs PTNL; PTL mean relative expression: 65.89 ± 31.65; TL mean relative expression: 5.95 ± 1.69; PTNL mean relative expression: 3.02 ± 0.85; Fig. 5C).

Given that the microarray analysis identified *FGA, FGB* and *FGG* to be among the top up-regulated genes in PTL decidual samples, and that KEGG pathway enrichment analysis identified ‘complement and coagulation cascades’ to be a significantly enriched pathway in PTL, we also examined the expression of several key genes involved in coagulation by qRT-PCR. We found that although the mean concentrations of *FGA* (PTL mean relative expression: 30.38 ± 16.14; TL mean relative expression: 2.23 ± 1.05; Fig. 5D), *FGB* (PTL mean relative expression: 10.98 ± 4.51; TL mean relative expression: 2.03 ± 0.54; Fig. 5E) and *FGG* mRNAs (PTL mean relative expression: 7.85 ± 3.39; TL mean relative expression: 1.17 ± 0.29; Fig. 5F) were all elevated in PTL decidua samples, but this increase did not reach statistical significance (13.6-fold, 5.4-fold; and 6.7-fold increased expression vs TL samples, *P* = 0.21, *P* = 0.36 and *P* = 0.06, respectively) due to variations between samples (e.g. Fig. 5D). Similarly, the significantly elevated expression of tissue plasminogen activator, *PLAT*, observed in the microarray analysis was not validated by qRT-PCR (2.3-fold increase PTL vs. TL, *P* = 0.19; PTL mean relative expression: 3.98 ± 0.69; TL mean relative expression: 1.75 ± 0.40; Fig. 5G). However, expression of the urokinase-type plasminogen activator, *PLAU*, was significantly greater in PTL decidua samples, compared with TL samples (2-fold, *P* < 0.05; PTL mean relative expression: 2.34 ± 0.34; TL mean relative expression: 1.16 ± 0.33; Fig. 5H).

## Discussion

The decidua, situated directly at the maternal–foetal interface, is ideally located to play a pivotal role in the onset of parturition, both at term and preterm. However, compared to other gestational tissues (myometrium, foetal membranes and cervix), the exact role the decidua plays in the onset of labour remains relatively poorly understood. In this study, we describe the lymphocyte milieu of the human decidua and examine for the first time the decidual transcriptome in both normal physiological labour at term and in pathological PTL. We found no changes in any of the immune cell populations studied (NK cells, B cells, T cells and iNKT cells) in association with parturition, but an up-regulation in inflammatory gene expression in association with term and preterm parturition and of the iNKT cell activation marker CD1D in association with PTL.

In agreement with previous work (Sindram-Trujillo *et al.*, 2004; Williams *et al.*, 2009; Gomez-Lopez *et al.*, 2013; Bartmann *et al.*, 2014), we found that the decidua, both at term and preterm, hosts a variety of lymphocytes, including NK cells, T cells, B cells and iNKT cells, of which T cells and NK cells made up the largest proportions of the decidual CD45^+^ lymphocytes. There are conflicting reports on labour- and gestation-associated changes in overall leucocyte density in the decidua, with some studies reporting an increase in the CD45^+^ population at term compared to preterm (Gomez-Lopez *et al.*, 2013; Bartmann *et al.*, 2014), whilst another study found no difference in the decidual CD45^+^ population in women at term, either in labour or not in labour, or in women in PTL (Hamilton *et al.*, 2012). The results in the current study were in agreement with Hamilton *et al.* (2012), with no significant difference in the number of CD45^+^ lymphocytes between decidua recovered from our four patient groups. Similarly, there is little consensus on changes to decidual T cell number, with one study reporting elevated T cell number at term, compared to preterm (Gomez-Lopez *et al.*, 2013), which contrasts another study reporting a significant increase in CD3^+^ T cells in PTL samples compared to TL samples (Hamilton *et al.*, 2012). Hamilton *et al.* (2012) also report a significant increase in CD56^+^ NK cells in decidua from women in PTL compared to TL, however we found no significant difference in decidual T cell or NK cell populations in our sample groups (TNL, TL, PTNL and PTL).

The lack of agreement between the different studies highlights the difficulty in characterizing changes in decidual immune cell populations, as in each study there is quite high inter-patient variability. Given the limited number of studies that have investigated the decidual immune cell populations in relation to parturition, particularly in PTL, confirmatory studies with larger sample sizes would be helpful. Our preterm sample sizes were particularly limited not only due to the difficulty in obtaining preterm samples but also because of the limited availability of decidual tissue present on the preterm foetal membranes. Therefore, some samples did not provide a large enough decidual leucocyte population for flow cytometry analysis. The differences in the results presented here and in previously published studies (where labour-associated differences in immune cell populations were observed) may be explained by methodological differences. Both our study and that by Gomez-Lopez *et al.* (2013) used flow cytometry analysis, which allowed us to quantify and stain the whole panel of lymphocytes present in our decidual samples. In contrast, Hamilton *et al.* (2012) took an immunohistochemical approach with manual counting of a fixed number of fields of view. Furthermore, our decidual samples were collected by carefully scraping decidua parietalis from the chorion layer of the foetal membranes, allowing us to specifically analyse the decidua parietalis lymphocyte populations. However, in the other studies the chorio-decidua together or decidua basalis was examined. Although both decidua basalis and decidua parietalis are part of the decidual tissue, they are composed of different cell types and it has previously been shown that there is differential distribution of NK cells and T cells at term between the two decidual tissue sites (Sindram-Trujillo *et al.*, 2003a, b). Therefore, it is important to note that the observations in our study are specific to the decidua parietalis and there may be alterations in lymphocyte cell number in the decidua basalis in association with term and PTL, which we have not studied here. We believe that our study, in which we have focussed on the decidua parietalis only and used the gold standard method of flow cytometry to count cells, is robust, and that our finding that there is no difference in decidual cell lymphocyte cell density in association with parturition (at least in the decidua parietalis) is likely to be reproducible.

It should also be noted that we have only examined the proportion of each lymphocyte population in this study and have not measured the activation status of these lymphocytes. The ability of leucocytes, present in gestational tissues at the time of parturition, to contribute to the inflammatory response by producing pro-inflammatory cytokines, chemokines and MMPs has been well described (Roh *et al.*, 2000; Young *et al.*, 2002; Gomez-Lopez *et al.*, 2013). Future studies examining whether these decidual lymphocytes are activated and producing pro-inflammatory mediators will give further information on whether they provide a functional role in the labour process either at term or preterm.

In recent years, decidual iNKT cells have become an immune cell subpopulation of particular interest in the context of PTB (Rinaldi *et al.*, 2015). The presence of iNKT cells in both the pregnant human and mouse decidua has been established (Ito *et al.*, 2000; Tsuda *et al.*, 2001; Boyson *et al.*, 2002; St Louis *et al.*, 2016; Negishi *et al.*, 2017). Animal studies have suggested a key role for iNKT cells in driving PTL, where iNKT cell activation has been shown to result in both early pregnancy loss and PTB in the mouse (Ito *et al.*, 2000; Boyson *et al.*, 2002; Li *et al.*, 2015; St Louis *et al.*, 2016). Furthermore, Jα18 knockout mice, which lack iNKT cells, have been shown to have a reduced rate of lipopolysaccharide (LPS)-induced early pregnancy loss and PTB (Li *et al.*, 2012; 2013). These studies provided compelling evidence that iNKT cells likely play an important pathological role in PTL in mice, and a recent study has reported that iNKT cell number is higher in decidual samples collected from women in PTB without chorioamnionitis compared with PTB with chorioamnionitis (Negishi *et al.*, 2017). Prior to the data shown here, no other studies had investigated whether the proportions of decidual iNKT cells are altered in human labour both at term and preterm.

In this study, we identified a small population of CD3^+^ TCRVα24-Jα18 + iNKT cells in the decidua samples collected from each of our four patient groups, which accounted for around 0.1% of the decidual CD3^+^ T cell population. Reports of third trimester decidual iNKT cell numbers are limited, with only two other studies having recently published these data (St Louis *et al.*, 2016; Negishi *et al.*, 2017), although one study only examined ‘iNKT-like’ cells and the other study only examined iNKT cells in women with late PTB (34–36 weeks). In contrast to our findings of no difference in decidual iNKT cell number between our four patient groups, both studies report elevated numbers of ‘iNKT-like’ and iNKT cells in decidual samples from women with non-infection-associated PTL. Due to differences in study design, patient inclusion criteria and iNKT cell markers used, it is difficult to make direct comparisons across the three studies, however given our use of antibodies against the specific iNKT cell marker in our study (TCRVα24-Jα18) and our inclusion of our four patient groups (TNL, TL, PTNL and PTL), we believe our data more accurately reflects the true proportion of iNKT cells in third trimester decidua in both labour and PTL. Future studies with larger sample sizes are required to further investigate decidual iNKT cell numbers in PTL.

Interestingly, although we did not observe a difference in iNKT cell number in PTL decidua, we found increased expression of the non-classical MHC-protein, CD1d at both the mRNA and protein level in decidual samples collected from patients in PTL. CD1d is involved in iNKT cell activation via lipid antigen presentation (Brennan *et al.*, 2013), and is expressed on professional-antigen presenting cells (APCs), including macrophages, dendritic cells, B cells, activated T cells (Exley *et al.*, 2000; Brigl and Brenner, 2004) and intestinal epithelial cells (Blumberg *et al.*, 1991). Decidual CD1d expression has been reported on both villous and extravillous trophoblast cells (Boyson *et al.*, 2002; Matsumoto *et al.*, 2008) and its expression increases as gestation progresses (Shao *et al.*, 2005). To the best of our knowledge, this is the first study to examine CD1d expression in third trimester decidual samples collected from TNL, TL, PTNL and PTL women, and the first study to report elevated decidual CD1d expression in association with PTL.

The reason for the greater CD1d expression in our PTL samples (compared to TNL and PTNL) is currently unclear, but it has been previously shown that CD1d expression on APC can be induced by inflammatory cytokines, such as IFN-γ and TNF or in the presence of bacteria (Skold *et al.*, 2005). Given that 50% of our PTL samples had confirmed chorioamnionitis (as might be expected), and that we showed increased expression of a number of inflammatory mediators in PTL decidua samples (according to our microarray results), it is possible that this mechanism explains the elevated CD1d observed in our samples. Interestingly, this finding suggests that although we did not see an increase in the proportion of decidual iNKT cells present in PTL decidua samples, the increased CD1d expression may result in more activated iNKT cells in PTL decidua, which could go on to stimulate other immune cells and contribute to the pathological inflammatory response associated with PTL. In support of this hypothesis, St Louis *et al.* (2016) reported the presence of activated iNKT cells (CD3^+^ Vα24 Jα18TCR + CD69^+^ cells) in PTL and TL decidua samples (St Louis *et al.*, 2016), but no formal quantification of these activated cells was reported. Further work is required to confirm this observation, but our results along with that of St Louis *et al.* (2016), suggest that the relationship between decidual iNKT cells and CD1d in PTL warrants further investigation.

To complement the analysis of decidual immune cell populations, we also investigated the decidual transcriptome in our four patient groups. Although several genome-wide microarray studies have been carried out to improve our understanding of the gene expression changes associated with the onset of labour, the decidua has been largely neglected compared to other gestational tissues (Eidem *et al.*, 2015). To date, only two other transcriptomic studies have reported examining the labour-associated gene expression changes in the human decidua, with one studying focusing on term labour (Stephen *et al.*, 2015) and the other study focussing on PTL (Shankar *et al.*, 2010). Our microarray data presented here complements and extends these studies, by examining the gene expression signature of the decidua during both term and PTL.

Our findings are broadly in agreement with the study of Stephen *et al.* (2015), demonstrating that term labour is associated with extensive inflammatory activation, as has been established for other gestational tissues such as the myometrium, foetal membranes and cervix (Bollapragada *et al.*, 2009; Mittal *et al.*, 2010; Weiner *et al.*, 2010; Sharp *et al.*, 2016). KEGG pathway analysis revealed that several inflammatory and immune pathways were enriched in TL samples, including ‘TNF-signalling pathway’, ‘NOD-like receptor signalling pathway’, ‘NF-kappa B signalling pathway’ and ‘Toll like receptor signalling pathway’. Additionally, our qRT-PCR results validated the finding that a number of important inflammatory genes were significantly up-regulated in TL samples compared with TNL decidua. Notably elevated expression of genes with an established role in parturition (such as *IL-6* and *PTGS2*) is in line with expectations (Keelan *et al.*, 2003; Osman *et al.*, 2006; Bollapragada *et al.*, 2009). We also describe, for the first time, that the decidua from women in TL has elevated expression of a number of genes that are involved in negatively regulating inflammation, namely *IER3, TNFAIP3 and ATF3.* This may suggest that whilst the decidua contributes to the pro-inflammatory signalling associated with the onset of parturition, it may also have an important role in maintaining inflammatory homoeostasis during physiological term labour. In support of this hypothesis, a recent study investigating inflammatory gene networks in term human decidual stromal cells stimulated with IL-1β *in vitro* identified the up-regulation of several microRNAs that regulate pro-inflammatory gene expression (Ibrahim *et al.*, 2016).

To determine whether there was a specific decidual gene signature associated with PTL we also compared gene expression in PTL samples with both TL and PTNL samples. Our network graph analysis demonstrated that the PTL samples appeared to have the most similar gene expression, with 75% of these samples clustering together. This analysis, followed by our KEGG pathway enrichment analysis and qRT-PCR validations have led us to identify several genes of interest that are significantly elevated in PTL samples.

KEGG pathway analysis identified that one of the top enriched pathways in our PTL samples was ‘complement and coagulation cascades’. Such a link has been reported in other studies (Bogic *et al.*, 1999; Elovitz *et al.*, 2001; Norwitz *et al.*, 2007; Velez *et al.*, 2008; Shankar *et al.*, 2010; Phillippe *et al.*, 2011), although with PCR we were unable to validate up-regulation of complement and coagulation associated genes: *FGA, FGB, FGG* and *PLAT*, which we showed in the microarray. We did validate elevated expression of *PLAU*, which encodes for urokinase plasminogen activator (uPA). uPA is a serine protease which is involved in converting inactive plasminogen to plasmin and can regulate ECM degradation by activating MMPs (Vassalli *et al.*, 1991; Baricos *et al.*, 1995; Zhao *et al.*, 2008). In gestational tissues, high uPA expression has been reported in the decidual cells adjacent to the area of foetal membrane rupture in humans and rhesus monkeys (Liu *et al.*, 1998). To the best of our knowledge, we believe this is the first study to report elevated expression of *PLAU* in decidua collected from women in PTL. We know that labour is associated with significant ECM remodelling and MMP activity within the decidua (Ulug *et al.*, 2001; Li *et al.*, 2004); therefore given the link between uPA, plasmin and MMP activity, it is possible that this elevated *PLAU* expression in PTL may promote premature ECM degradation and foetal membrane rupture, resulting in the onset of PTL.

Another important pathway which was highlighted by our KEGG analysis to be significantly up-regulated in PTL was ‘cytokine–cytokine receptor interaction’, suggesting that inflammatory signalling also plays a role in the pathogenesis of PTL, as observed in TL. Indeed, we validated the mRNA expression of several genes that are linked to inflammation by qRT-PCR which were significantly elevated only in PTL decidua samples, *CXCL8*, *MARCO*, and *LILRA3.* Of these three genes, only *CXCL8* has been previously linked to parturition (Romero *et al.*, 1991; Kelly *et al.*, 1992; Osmers *et al.*, 1995; Denison *et al.*, 1998; Tornblom *et al.*, 2005; Gomez-Lopez *et al.*, 2009; Hamilton *et al.*, 2013; Stephen *et al.*, 2015). The other two inflammation-related genes, *MARCO* and *LILRA3*, have not previously been linked to PTL, and could be interesting novel targets for therapeutic investigation. MARCO (macrophage receptor with collagenous structure) is a class A scavenger receptor encoded by the *MARCO* gene, which is constitutively expressed by subsets of macrophages and has been shown to be up-regulated in the presence of infection or in inflammatory conditions *in vivo* (van der Laan *et al.*, 1999; Seta *et al.*, 2001; Milne *et al.*, 2005). MARCO has been detected in human and mouse decidual macrophages, where it has been reported to have an important role in the clearance of intrauterine infection with *Clostridium sordellii* (Thelen *et al.*, 2010). *LILRA3* (leucocyte immunoglobulin-like receptor-3), which encodes the LILRA3 protein, belongs to a family of LILR receptors, which have diverse activating and inhibitory actions on the immune response by altering the signalling of other immune receptors (Brown *et al.*, 2004). LILRA3 is secreted by immune cells, such as monocytes, mast cells, B cells and some subsets of T cells (Hirayasu and Arase, 2015). Only one other study has reported *LILRA3* expression in relation to parturition, where *LILRA3* expression was shown to be significantly down-regulated in the myometrium from women with dystocia, compared to women with normal uterine function (Brennan *et al.*, 2011); suggesting that LILRA3 may be involved in regulating the inflammatory and immune response which drives physiological labour at term. Hence, *MARCO* and *LILRA3*, which we link to PTL for the first time, have the potential to be important mediators in the pathological process of PTL and warrant further investigation.

Taken together, our decidual transcriptome studies demonstrate that there are widespread gene expression changes with labour onset, both at term and preterm, but importantly there are differences in the gene expression changes in TL and PTL. This finding agrees with the theory that PTL cannot simply be considered as labour that occurs too soon (Romero *et al.*, 2014). Although in both parturition processes we observed up-regulation of genes related to inflammatory processes, we were able to identify specific genes that were elevated only in TL or PTL. Therefore, using whole genome analysis, as we have done in this study, will help us to identify genes that are particularly involved in the pathological onset of PTL, which may help in the identification of novel therapeutic targets for PTB.

We believe the results presented in this study provide an important step forward in understanding the function of the decidua in both term and PTL as we have analysed the lymphocyte populations present within the decidua and carried out the first transcriptomic analysis using purified decidua tissue. However, we do recognize there are some limitations to our study. Our decidual immune cell analysis focused on the lymphocyte populations, therefore we are unable to comment on the contribution of decidual neutrophils or macrophages to TL or PTL in our samples, but these cell populations have been extensively examined elsewhere (Osman *et al.*, 2003; Hamilton *et al.*, 2012; Kim *et al.*, 2012; Shynlova *et al.*, 2013a, b). Another limitation to our study is that due to the difficulty in obtaining decidual samples, particularly from our preterm groups, it was necessary to perform the qRT-PCR validations on the same sample set. Additionally, our preterm samples (both PTL and PTNL) inevitably came from women with a range of underlying complications (e.g. chorioamnionitis, pre-eclampsia and IUGR), which may explain the high variability observed in these patient groups, because it is simply not possible to collect preterm samples from women with no underlying pathologies. This is a known limitation of studying PTL in women. Future studies, with very large sample sizes which can subgroup the preterm patients into different underlying pathologies may be more useful in identifying specific transcriptomic differences underlying the different aetiologies of PTL.

In summary, the data presented here demonstrate that the onset of parturition both at term and preterm is associated with widespread gene expression changes in the decidua, but not alterations in the number of lymphocytes present at the maternal–foetal interface (at least not in the sub-populations examined here). As well as reporting the elevated expression of pro-inflammatory genes, with known roles in labour, importantly we report, for the first time, the expression of genes involved in the regulation of the inflammatory response. Exploiting endogenous factors capable of regulating inflammation within gestational tissues may be an effective novel therapeutic target. Improving our understanding of the molecular mechanisms regulating the onset of labour both at term and preterm is critical to the development of new therapies for the treatment of PTB, which are urgently required. Increasing evidence suggests the decidua likely plays on important early role in the events triggering the onset of parturition, therefore, it is possible that targeting molecular changes occurring in the decidua may have more success than current therapies.

## Supplementary Material

Supplementary Data
