# Supplementary material for: Immune cell and transcriptomic analysis of the human decidua in term and preterm parturition
Source: Mol Hum Reprod. 2017 Aug 22;23(10):708–24. doi: 10.1093/molehr/gax038 (PMC5909855; doi:10.1093/molehr/gax038)
Supplement: Supplementary Data [file gax038_supplementarytables.docx]

**Supplementary Table I – Details of qRT-PCR Taqman Gene expression assays used**

| **Gene** | **Taqman gene expression assay number** |
| --- | --- |
| *ACTB* | 4310881E |
| *ATF3* | Hs00231069_m1 |
| *CXCL8* | Hs00174103_m1 |
| *FGA* | Hs00241027_m1 |
| *FGB* | Hs00241037_m1 |
| *FGG* | Hs00170586_m1 |
| *IER3* | Hs04187506_g1 |
| *IL-6* | Hs00985639_m1 |
| *LILRA3* | Hs00846590_s1 |
| *MARCO* | Hs00198935_m1 |
| *PLAT* | Hs00263492_m1 |
| *PLAU* | Hs01547054_m1 |
| *PTGS2* | HS00153133_m1 |
| *TNFAIP3* | Hs00234713_m1 |

**Supplementary Table II – Full list of differentially expressed genes identified by microarray analysis**

| **Comparison** | **Symbol** | **Description** | **Fold change** | ***p* Value** |
| --- | --- | --- | --- | --- |
| **TL vs. TNL** | *TNFAIP3* | tumor necrosis factor, alpha-induced protein 3 | 2.716 | 1.40E-04 |
|  | *IER3* | immediate early response 3 | 2.68 | 2.37E-03 |
|  | *IL6* | interleukin 6 | 2.657 | 8.79E-03 |
|  | *PTGS2* | prostaglandin-endoperoxide synthase 2 (prostaglandin G/H synthase and cyclooxygenase) | 2.474 | 2.15E-03 |
|  | *ATF3* | activating transcription factor 3 | 2.31 | 6.30E-03 |
|  | *PHLDA1* | pleckstrin homology-like domain, family A, member 1 | 2.147 | 3.98E-03 |
|  | *DUSP5* | dual specificity phosphatase 5 | 2.066 | 3.64E-03 |
|  | *NFKBIA* | nuclear factor of kappa light polypeptide gene enhancer in B-cells inhibitor, alpha | 1.988 | 8.04E-04 |
|  | *ZC3H12A* | zinc finger CCCH-type containing 12A | 1.905 | 3.84E-03 |
|  | *DDIT4* | DNA-damage-inducible transcript 4 | 1.824 | 4.79E-03 |
|  | *NEDD9* | neural precursor cell expressed, developmentally down-regulated 9 | 1.725 | 1.10E-04 |
|  | *GEM* | GTP binding protein overexpressed in skeletal muscle | 1.721 | 6.68E-03 |
|  | *PFKFB3* | 6-phosphofructo-2-kinase/fructose-2,6-biphosphatase 3 | 1.686 | 8.14E-03 |
|  | *HSPA1B* | heat shock 70kDa protein 1B | 1.673 | 1.55E-03 |
|  | *CSRNP1* | cysteine-serine-rich nuclear protein 1 | 1.668 | 5.34E-03 |
|  | *SIK1* | salt-inducible kinase 1 | 1.626 | 7.59E-03 |
|  | *MAFF* | v-maf avian musculoaponeurotic fibrosarcoma oncogene homolog F | 1.551 | 1.73E-03 |
|  | *CLK1* | CDC-like kinase 1 | 1.548 | 2.14E-05 |
|  | *MAP3K8* | mitogen-activated protein kinase kinase kinase 8 | 1.513 | 4.06E-03 |
|  | *ETS1* | v-ets avian erythroblastosis virus E26 oncogene homolog 1 | 1.445 | 1.75E-03 |
|  | *SLC20A1* | solute carrier family 20 (phosphate transporter), member 1 | 1.434 | 1.87E-03 |
|  | *EIF4A3* | eukaryotic translation initiation factor 4A3 | 1.429 | 8.26E-03 |
|  | *HNRNPA2B1* | heterogeneous nuclear ribonucleoprotein A2/B1 | 1.425 | 1.85E-04 |
|  | *ABL2* | ABL proto-oncogene 2, non-receptor tyrosine kinase | 1.393 | 9.24E-05 |
|  | *WTAP* | Wilms tumor 1 associated protein | 1.367 | 5.07E-03 |
|  | *PELI1* | pellino E3 ubiquitin protein ligase 1 | 1.358 | 3.24E-03 |
|  | *USP36* | ubiquitin specific peptidase 36 | 1.347 | 8.21E-04 |
|  | *FOSL1* | FOS-like antigen 1 | 1.343 | 7.20E-03 |
|  | *SOCS3* | suppressor of cytokine signaling 3 | 1.332 | 6.81E-03 |
|  | *SH3TC1* | SH3 domain and tetratricopeptide repeats 1 | 1.319 | 2.14E-03 |
|  | *VEZT* | vezatin, adherens junctions transmembrane protein | 1.296 | 2.49E-04 |
|  | *DDX41* | DEAD (Asp-Glu-Ala-Asp) box polypeptide 41 | 1.293 | 2.89E-03 |
|  | *NR4A3* | nuclear receptor subfamily 4, group A, member 3 | 1.286 | 3.85E-03 |
|  | *RNF165* | ring finger protein 165 | 1.286 | 5.92E-03 |
|  | *AK2* | adenylate kinase 2 | 1.272 | 8.35E-03 |
|  | *MIR21* | microRNA 21 | 1.262 | 7.21E-03 |
|  | *WDR33* | WD repeat domain 33 | 1.257 | 8.05E-03 |
|  | *DENND1A* | DENN/MADD domain containing 1A | 1.252 | 4.90E-03 |
|  | *SHB* | Src homology 2 domain containing adaptor protein B | 1.25 | 3.88E-03 |
|  | *PADI4* | peptidyl arginine deiminase, type IV | 1.248 | 8.54E-03 |
|  | *PRO0628* | uncharacterized LOC29053 | 1.246 | 7.12E-03 |
|  | *JMJD6* | jumonji domain containing 6 | 1.244 | 7.18E-03 |
|  | *CDKN2D* | cyclin-dependent kinase inhibitor 2D (p19, inhibits CDK4) | 1.241 | 2.66E-03 |
|  | *KDM6B* | lysine (K)-specific demethylase 6B | 1.24 | 3.88E-04 |
|  | *MLLT6* | myeloid/lymphoid or mixed-lineage leukemia (trithorax homolog, Drosophila); translocated to, 6 | 1.235 | 6.48E-03 |
|  | *RTN4R* | reticulon 4 receptor | 1.233 | 5.15E-03 |
|  | *HNRNPL* | heterogeneous nuclear ribonucleoprotein L | 1.23 | 1.58E-03 |
|  | *NHP2L1* | NHP2 non-histone chromosome protein 2-like 1 (S. cerevisiae) | 1.23 | 2.54E-03 |
|  | *RSC1A1* | regulatory solute carrier protein, family 1, member 1 | 1.219 | 8.74E-03 |
|  | *PLK3* | polo-like kinase 3 | 1.218 | 3.47E-03 |
|  | *LSMEM1* | leucine-rich single-pass membrane protein 1 | 1.217 | 9.80E-03 |
|  | *DHCR7* | 7-dehydrocholesterol reductase | 1.214 | 3.33E-03 |
|  | *SCAPER* | S-phase cyclin A-associated protein in the ER | 1.211 | 3.25E-03 |
|  | *RALGAPB* | Ral GTPase activating protein, beta subunit (non-catalytic) | 1.21 | 1.06E-03 |
|  | *PNISR* | PNN-interacting serine/arginine-rich protein | 1.206 | 1.26E-03 |
|  | *FOSL2* | FOS-like antigen 2 | 1.205 | 6.84E-03 |
|  | *MMP7* | matrix metallopeptidase 7 (matrilysin, uterine) | -2.387 | 1.84E-03 |
|  | *ZC3HAV1* | zinc finger CCCH-type, antiviral 1 | -1.564 | 1.51E-05 |
|  | *COL6A3* | collagen, type VI, alpha 3 | -1.538 | 4.49E-03 |
|  | *CIRBP* | cold inducible RNA binding protein | -1.456 | 1.88E-03 |
|  | *NID1* | nidogen 1 | -1.396 | 7.91E-03 |
|  | *CYB561D1* | cytochrome b561 family, member D1 | -1.394 | 2.64E-03 |
|  | *CLSTN1* | calsyntenin 1 | -1.355 | 9.51E-03 |
|  | *LHPP* | phospholysine phosphohistidine inorganic pyrophosphate phosphatase | -1.338 | 2.94E-03 |
|  | *RAB22A* | RAB22A, member RAS oncogene family | -1.327 | 9.02E-04 |
|  | *CCDC106* | coiled-coil domain containing 106 | -1.303 | 6.30E-03 |
|  | *CBX6* | chromobox homolog 6 | -1.3 | 4.38E-03 |
|  | *SRSF3* | serine/arginine-rich splicing factor 3 | -1.281 | 1.21E-04 |
|  | *RCN2* | reticulocalbin 2, EF-hand calcium binding domain | -1.271 | 6.25E-03 |
|  | *TMCO3* | transmembrane and coiled-coil domains 3 | -1.259 | 4.32E-03 |
|  | *APPBP2* | amyloid beta precursor protein (cytoplasmic tail) binding protein 2 | -1.257 | 5.69E-03 |
|  | *CENPB* | centromere protein B, 80kDa | -1.256 | 5.65E-04 |
|  | *FLJ42627* | uncharacterized LOC645644 | -1.253 | 3.80E-03 |
|  | *UHMK1* | U2AF homology motif (UHM) kinase 1 | -1.252 | 8.18E-03 |
|  | *MGAT2* | mannosyl (alpha-1,6-)-glycoprotein beta-1,2-N-acetylglucosaminyltransferase | -1.247 | 8.47E-03 |
|  | *FAM219B* | family with sequence similarity 219, member B | -1.247 | 2.69E-03 |
|  | *ELP5* | elongator acetyltransferase complex subunit 5 | -1.244 | 3.43E-03 |
|  | *SRP9* | signal recognition particle 9kDa | -1.243 | 7.26E-04 |
|  | *RNASEH1* | ribonuclease H1 | -1.242 | 1.58E-03 |
|  | *DCAF8* | DDB1 and CUL4 associated factor 8 | -1.238 | 4.81E-03 |
|  | *ANGEL2* | angel homolog 2 (Drosophila) | -1.238 | 8.85E-04 |
|  | *FYCO1* | FYVE and coiled-coil domain containing 1 | -1.238 | 8.13E-03 |
|  | *KLHDC2* | kelch domain containing 2 | -1.234 | 4.46E-03 |
|  | *FAF2* | Fas associated factor family member 2 | -1.233 | 4.17E-03 |
|  | *COA5* | cytochrome c oxidase assembly factor 5 | -1.23 | 4.83E-03 |
|  | *CES2* | carboxylesterase 2 | -1.229 | 5.66E-04 |
|  | *ZMAT3* | zinc finger, matrin-type 3 | -1.228 | 5.26E-03 |
|  | *MTCH1* | mitochondrial carrier 1 | -1.228 | 4.10E-03 |
|  | *SIVA1* | SIVA1, apoptosis-inducing factor | -1.226 | 7.74E-03 |
|  | *SNX1* | sorting nexin 1 | -1.226 | 1.29E-04 |
|  | *WRB* | tryptophan rich basic protein | -1.226 | 4.08E-03 |
|  | *NMT2* | N-myristoyltransferase 2 | -1.225 | 5.14E-03 |
|  | *ATP2A2* | ATPase, Ca++ transporting, cardiac muscle, slow twitch 2 | -1.222 | 3.85E-03 |
|  | *CS* | citrate synthase | -1.219 | 1.70E-03 |
|  | *YIF1A* | Yip1 interacting factor homolog A (S. cerevisiae) | -1.218 | 9.11E-03 |
|  | *PIGQ* | phosphatidylinositol glycan anchor biosynthesis, class Q | -1.213 | 2.08E-03 |
|  | *NDUFS8* | NADH dehydrogenase (ubiquinone) Fe-S protein 8, 23kDa (NADH-coenzyme Q reductase) | -1.212 | 7.10E-03 |
|  | *TLN1* | talin 1 | -1.21 | 3.56E-03 |
|  | *TM2D3* | TM2 domain containing 3 | -1.21 | 6.87E-03 |
|  | *OXLD1* | oxidoreductase-like domain containing 1 | -1.209 | 4.54E-03 |
|  | *RBBP7* | retinoblastoma binding protein 7 | -1.205 | 1.48E-03 |
|  | *STAU1* | staufen double-stranded RNA binding protein 1 | -1.204 | 1.20E-03 |
|  | *HDDC2* | HD domain containing 2 | -1.202 | 7.36E-03 |
|  | *TIMM21* | translocase of inner mitochondrial membrane 21 homolog (yeast) | -1.201 | 9.91E-04 |
| **TL vs. PTL** | *FOSB* | FBJ murine osteosarcoma viral oncogene homolog B | 2.538 | 9.45E-03 |
|  | *FST* | follistatin | 2.325 | 3.96E-03 |
|  | *HES4* | hes family bHLH transcription factor 4 | 1.691 | 9.09E-04 |
|  | *KLF4* | Kruppel-like factor 4 (gut) | 1.563 | 9.62E-03 |
|  | *SASH1* | SAM and SH3 domain containing 1 | 1.493 | 8.10E-03 |
|  | *HRK* | harakiri, BCL2 interacting protein | 1.492 | 9.84E-03 |
|  | *NEDD9* | neural precursor cell expressed, developmentally down-regulated 9 | 1.489 | 5.24E-03 |
|  | *ELMSAN1* | ELM2 and Myb/SANT-like domain containing 1 | 1.44 | 2.50E-03 |
|  | *FOXC1* | forkhead box C1 | 1.418 | 4.11E-03 |
|  | *CCNB1IP1* | cyclin B1 interacting protein 1, E3 ubiquitin protein ligase | 1.414 | 6.07E-03 |
|  | *WDR33* | WD repeat domain 33 | 1.403 | 7.22E-03 |
|  | *RNF165* | ring finger protein 165 | 1.391 | 1.08E-03 |
|  | *TGFBR3* | transforming growth factor, beta receptor III | 1.37 | 9.66E-03 |
|  | *SUN1* | Sad1 and UNC84 domain containing 1 | 1.356 | 6.62E-03 |
|  | *SNORA28* | small nucleolar RNA, H/ACA box 28 | 1.349 | 9.21E-04 |
|  | *MPC1* | mitochondrial pyruvate carrier 1 | 1.299 | 3.36E-03 |
|  | *FUBP3* | far upstream element (FUSE) binding protein 3 | 1.276 | 1.19E-03 |
|  | *RAD23B* | RAD23 homolog B (S. cerevisiae) | 1.273 | 2.26E-04 |
|  | *FAM168B* | family with sequence similarity 168, member B | 1.27 | 9.29E-03 |
|  | *PPP1R3B* | protein phosphatase 1, regulatory subunit 3B | 1.27 | 4.02E-03 |
|  | *DDX41* | DEAD (Asp-Glu-Ala-Asp) box polypeptide 41 | 1.27 | 8.88E-03 |
|  | *ZHX3* | zinc fingers and homeoboxes 3 | 1.269 | 7.35E-03 |
|  | *SIAH2* | siah E3 ubiquitin protein ligase 2 | 1.253 | 7.61E-03 |
|  | *NHP2L1* | NHP2 non-histone chromosome protein 2-like 1 (S. cerevisiae) | 1.245 | 2.94E-03 |
|  | *CDKN1B* | cyclin-dependent kinase inhibitor 1B (p27, Kip1) | 1.24 | 4.47E-03 |
|  | *LAMP2* | lysosomal-associated membrane protein 2 | 1.238 | 1.69E-04 |
|  | *BEX2* | brain expressed X-linked 2 | 1.229 | 5.25E-03 |
|  | *CCDC59* | coiled-coil domain containing 59 | 1.228 | 6.75E-03 |
|  | *RPS6* | ribosomal protein S6 | 1.225 | 6.30E-03 |
|  | *BRIX1* | BRX1, biogenesis of ribosomes, homolog (S. cerevisiae) | 1.219 | 1.53E-03 |
|  | *AKAP12* | A kinase (PRKA) anchor protein 12 | 1.211 | 1.65E-04 |
|  | *UPF3A* | UPF3 regulator of nonsense transcripts homolog A (yeast) | 1.21 | 4.02E-03 |
|  | *FGA* | fibrinogen alpha chain | -4.108 | 6.28E-03 |
|  | *FGG* | fibrinogen gamma chain | -3.688 | 5.88E-04 |
|  | *FGB* | fibrinogen beta chain | -2.753 | 2.53E-03 |
|  | *PLAT* | plasminogen activator, tissue | -2.235 | 5.73E-03 |
|  | *MARCO* | macrophage receptor with collagenous structure | -2.087 | 4.03E-03 |
|  | *LILRA3* | leukocyte immunoglobulin-like receptor, subfamily A (without TM domain), member 3 | -1.945 | 9.45E-03 |
|  | *NR1H3* | nuclear receptor subfamily 1, group H, member 3 | -1.769 | 8.67E-03 |
|  | *KCNK4* | potassium channel, subfamily K, member 4 | -1.75 | 1.95E-03 |
|  | *TNFRSF8* | tumor necrosis factor receptor superfamily, member 8 | -1.72 | 1.53E-03 |
|  | *WARS* | tryptophanyl-tRNA synthetase | -1.712 | 9.12E-03 |
|  | *TNFSF14* | tumor necrosis factor (ligand) superfamily, member 14 | -1.68 | 1.24E-03 |
|  | *TSPO* | translocator protein (18kDa) | -1.679 | 8.73E-04 |
|  | *NLGN4X* | neuroligin 4, X-linked | -1.63 | 7.49E-03 |
|  | *CDC7* | cell division cycle 7 | -1.578 | 1.36E-03 |
|  | *THY1* | Thy-1 cell surface antigen | -1.549 | 5.09E-03 |
|  | *TMEM176A* | transmembrane protein 176A | -1.511 | 6.76E-03 |
|  | *HK3* | hexokinase 3 (white cell) | -1.483 | 8.49E-03 |
|  | *PLD1* | phospholipase D1, phosphatidylcholine-specific | -1.458 | 6.21E-03 |
|  | *SLC2A6* | solute carrier family 2 (facilitated glucose transporter), member 6 | -1.453 | 6.16E-03 |
|  | *DRAM1* | DNA-damage regulated autophagy modulator 1 | -1.452 | 3.62E-03 |
|  | *LILRA6* | leukocyte immunoglobulin-like receptor, subfamily A (with TM domain), member 6 | -1.445 | 9.44E-03 |
|  | *MOSPD2* | motile sperm domain containing 2 | -1.419 | 9.97E-03 |
|  | *JDP2* | Jun dimerization protein 2 | -1.413 | 1.83E-03 |
|  | *STOM* | stomatin | -1.403 | 7.84E-03 |
|  | *STEAP3* | STEAP family member 3, metalloreductase | -1.378 | 2.69E-03 |
|  | *ATF5* | activating transcription factor 5 | -1.372 | 7.68E-03 |
|  | *TLR4* | toll-like receptor 4 | -1.365 | 8.34E-03 |
|  | *CPD* | carboxypeptidase D | -1.365 | 7.93E-03 |
|  | *DISC1* | disrupted in schizophrenia 1 | -1.361 | 9.25E-03 |
|  | *HOXA11-AS* | HOXA11 antisense RNA | -1.356 | 5.95E-03 |
|  | *HIST1H2BG* | histone cluster 1, H2bg | -1.346 | 8.62E-03 |
|  | *NDST2* | N-deacetylase/N-sulfotransferase (heparan glucosaminyl) 2 | -1.344 | 1.35E-04 |
|  | *MARCH2* | membrane-associated ring finger (C3HC4) 2, E3 ubiquitin protein ligase | -1.342 | 3.68E-03 |
|  | *PARP12* | poly (ADP-ribose) polymerase family, member 12 | -1.331 | 8.68E-03 |
|  | *TYMP* | thymidine phosphorylase | -1.33 | 9.49E-03 |
|  | *BID* | BH3 interacting domain death agonist | -1.329 | 2.43E-03 |
|  | *PPP1R10* | protein phosphatase 1, regulatory subunit 10 | -1.327 | 7.57E-03 |
|  | *SPIDR* | scaffolding protein involved in DNA repair | -1.326 | 4.35E-03 |
|  | *CAPN3* | calpain 3, (p94) | -1.316 | 7.50E-03 |
|  | *SLC5A3* | solute carrier family 5 (sodium/myo-inositol cotransporter), member 3 | -1.313 | 1.00E-04 |
|  | *ELOVL6* | ELOVL fatty acid elongase 6 | -1.308 | 5.72E-03 |
|  | *YBEY* | ybeY metallopeptidase (putative) | -1.295 | 3.33E-03 |
|  | *GNS* | glucosamine (N-acetyl)-6-sulfatase | -1.293 | 1.98E-04 |
|  | *NADK* | NAD kinase | -1.292 | 9.75E-03 |
|  | *TRIM13* | tripartite motif containing 13 | -1.292 | 5.73E-04 |
|  | *AGA* | aspartylglucosaminidase | -1.29 | 7.04E-03 |
|  | *ACTR1A* | ARP1 actin-related protein 1 homolog A, centractin alpha (yeast) | -1.279 | 3.09E-03 |
|  | *TTC38* | tetratricopeptide repeat domain 38 | -1.273 | 7.38E-03 |
|  | *ATG7* | autophagy related 7 | -1.271 | 8.61E-03 |
|  | *TMEM44* | transmembrane protein 44 | -1.269 | 6.10E-03 |
|  | *G6PD* | glucose-6-phosphate dehydrogenase | -1.269 | 8.47E-03 |
|  | *DTX2* | deltex 2, E3 ubiquitin ligase | -1.262 | 1.61E-03 |
|  | *ATP6V0B* | ATPase, H+ transporting, lysosomal 21kDa, V0 subunit b | -1.255 | 8.48E-03 |
|  | *ATP6V1B2* | ATPase, H+ transporting, lysosomal 56/58kDa, V1 subunit B2 | -1.251 | 4.97E-03 |
|  | *ACOT9* | acyl-CoA thioesterase 9 | -1.248 | 3.84E-03 |
|  | *TLN1* | talin 1 | -1.246 | 1.98E-03 |
|  | *HIST2H2BF* | histone cluster 2, H2bf | -1.244 | 1.03E-03 |
|  | *PGR* | progesterone receptor | -1.241 | 7.22E-03 |
|  | *IL13RA1* | interleukin 13 receptor, alpha 1 | -1.241 | 9.65E-03 |
|  | *CSTF3* | cleavage stimulation factor, 3' pre-RNA, subunit 3, 77kDa | -1.236 | 6.97E-03 |
|  | *MYL6* | myosin, light chain 6, alkali, smooth muscle and non-muscle | -1.234 | 3.62E-03 |
|  | *PSMC3* | proteasome (prosome, macropain) 26S subunit, ATPase, 3 | -1.232 | 9.95E-03 |
|  | *COL22A1* | collagen, type XXII, alpha 1 | -1.227 | 6.35E-03 |
|  | *STAU1* | staufen double-stranded RNA binding protein 1 | -1.226 | 9.93E-04 |
|  | *HECTD3* | HECT domain containing E3 ubiquitin protein ligase 3 | -1.225 | 1.73E-04 |
|  | *RAB13* | RAB13, member RAS oncogene family | -1.222 | 8.27E-03 |
|  | *AK1* | adenylate kinase 1 | -1.222 | 1.65E-03 |
|  | *PPP2R4* | protein phosphatase 2A activator, regulatory subunit 4 | -1.221 | 9.72E-04 |
|  | *APTX* | aprataxin | -1.221 | 1.38E-03 |
|  | *LRCH4* | leucine-rich repeats and calponin homology (CH) domain containing 4 | -1.219 | 3.54E-03 |
|  | *TMEM69* | transmembrane protein 69 | -1.215 | 5.95E-04 |
|  | *DHFRL1* | dihydrofolate reductase-like 1 | -1.211 | 5.72E-03 |
|  | *CD81* | CD81 molecule | -1.211 | 5.71E-04 |
|  | *TXN* | thioredoxin | -1.21 | 1.99E-03 |
|  | *ITIH5* | inter-alpha-trypsin inhibitor heavy chain family, member 5 | -1.21 | 2.01E-03 |
|  | *DBNL* | drebrin-like | -1.206 | 9.22E-03 |
|  | *SLC39A3* | solute carrier family 39 (zinc transporter), member 3 | -1.205 | 6.47E-03 |
|  | *ZNF207* | zinc finger protein 207 | -1.205 | 2.99E-03 |
|  | *CSNK1G1* | casein kinase 1, gamma 1 | -1.204 | 8.64E-04 |
|  | *MSH3* | mutS homolog 3 | -1.201 | 2.97E-05 |
| **PTL vs. PTNL** | *CXCL8* | chemokine (C-X-C motif) ligand 8 | 5.441 | 2.49E-03 |
|  | *CEMIP* | cell migration inducing protein, hyaluronan binding | 3.338 | 6.97E-03 |
|  | *S100A8* | S100 calcium binding protein A8 | 3.193 | 1.02E-03 |
|  | *FGG* | fibrinogen gamma chain | 3.023 | 3.76E-03 |
|  | *SOD2* | superoxide dismutase 2, mitochondrial | 2.89 | 2.04E-03 |
|  | *FGB* | fibrinogen beta chain | 2.784 | 3.08E-03 |
|  | *NAMPT* | nicotinamide phosphoribosyltransferase | 2.742 | 1.03E-03 |
|  | *S100A9* | S100 calcium binding protein A9 | 2.547 | 9.93E-04 |
|  | *AQP9* | aquaporin 9 | 2.528 | 3.60E-03 |
|  | *IL1RN* | interleukin 1 receptor antagonist | 2.385 | 8.79E-03 |
|  | *PI3* | peptidase inhibitor 3, skin-derived | 2.257 | 3.40E-03 |
|  | *LILRA3* | leukocyte immunoglobulin-like receptor, subfamily A (without TM domain), member 3 | 2.189 | 3.62E-03 |
|  | *CSF3R* | colony stimulating factor 3 receptor (granulocyte) | 2.148 | 2.52E-03 |
|  | *MCEMP1* | mast cell-expressed membrane protein 1 | 2.105 | 5.20E-03 |
|  | *SIGLEC14* | sialic acid binding Ig-like lectin 14 | 2.075 | 1.61E-03 |
|  | *CHI3L1* | chitinase 3-like 1 (cartilage glycoprotein-39) | 2.054 | 7.83E-03 |
|  | *HSPA1A* | heat shock 70kDa protein 1A | 2.043 | 5.84E-03 |
|  | *SLC11A1* | solute carrier family 11 (proton-coupled divalent metal ion transporter), member 1 | 2.035 | 9.43E-03 |
|  | *MARCO* | macrophage receptor with collagenous structure | 2.003 | 8.01E-03 |
|  | *NOV* | nephroblastoma overexpressed | 1.984 | 6.13E-03 |
|  | *PDPN* | podoplanin | 1.975 | 1.86E-03 |
|  | *LILRA5* | leukocyte immunoglobulin-like receptor, subfamily A (with TM domain), member 5 | 1.897 | 4.67E-03 |
|  | *IL7R* | interleukin 7 receptor | 1.88 | 5.99E-03 |
|  | *SLC39A14* | solute carrier family 39 (zinc transporter), member 14 | 1.869 | 4.05E-03 |
|  | *SERPINA1* | serpin peptidase inhibitor, clade A (alpha-1 antiproteinase, antitrypsin), member 1 | 1.856 | 8.75E-03 |
|  | *TFRC* | transferrin receptor | 1.845 | 2.64E-04 |
|  | *C10orf10* | chromosome 10 open reading frame 10 | 1.844 | 2.69E-03 |
|  | *IRAK3* | interleukin-1 receptor-associated kinase 3 | 1.838 | 3.10E-04 |
|  | *NNMT* | nicotinamide N-methyltransferase | 1.83 | 6.61E-03 |
|  | *TUBB3* | tubulin, beta 3 class III | 1.83 | 2.50E-04 |
|  | *SLC16A10* | solute carrier family 16 (aromatic amino acid transporter), member 10 | 1.817 | 8.28E-04 |
|  | *TNFSF14* | tumor necrosis factor (ligand) superfamily, member 14 | 1.758 | 7.59E-04 |
|  | *HSPA1B* | heat shock 70kDa protein 1B | 1.729 | 2.32E-03 |
|  | *PAPSS2* | 3'-phosphoadenosine 5'-phosphosulfate synthase 2 | 1.725 | 1.80E-03 |
|  | *CNTNAP1* | contactin associated protein 1 | 1.724 | 5.12E-04 |
|  | *GK* | glycerol kinase | 1.706 | 5.69E-03 |
|  | *SLC2A6* | solute carrier family 2 (facilitated glucose transporter), member 6 | 1.644 | 6.78E-04 |
|  | *TNFRSF6B* | tumor necrosis factor receptor superfamily, member 6b, decoy | 1.619 | 5.04E-03 |
|  | *ACSL1* | acyl-CoA synthetase long-chain family member 1 | 1.59 | 2.33E-03 |
|  | *CCR7* | chemokine (C-C motif) receptor 7 | 1.556 | 8.83E-03 |
|  | *GPR84* | G protein-coupled receptor 84 | 1.556 | 5.00E-03 |
|  | *TMEM176A* | transmembrane protein 176A | 1.555 | 5.34E-03 |
|  | *PLD1* | phospholipase D1, phosphatidylcholine-specific | 1.539 | 2.82E-03 |
|  | *HK3* | hexokinase 3 (white cell) | 1.519 | 7.07E-03 |
|  | *EIF4A3* | eukaryotic translation initiation factor 4A3 | 1.484 | 8.62E-03 |
|  | *PCNX* | pecanex homolog (Drosophila) | 1.47 | 3.05E-03 |
|  | *BCL6* | B-cell CLL/lymphoma 6 | 1.463 | 7.71E-03 |
|  | *GSTO1* | glutathione S-transferase omega 1 | 1.448 | 1.29E-03 |
|  | *DRAM1* | DNA-damage regulated autophagy modulator 1 | 1.444 | 5.32E-03 |
|  | *DCUN1D3* | DCN1, defective in cullin neddylation 1, domain containing 3 | 1.44 | 4.12E-03 |
|  | *PI15* | peptidase inhibitor 15 | 1.435 | 3.77E-03 |
|  | *GCA* | grancalcin, EF-hand calcium binding protein | 1.425 | 8.73E-03 |
|  | *SLC5A3* | solute carrier family 5 (sodium/myo-inositol cotransporter), member 3 | 1.424 | 3.84E-06 |
|  | *VWA1* | von Willebrand factor A domain containing 1 | 1.418 | 7.60E-03 |
|  | *ATF5* | activating transcription factor 5 | 1.41 | 5.32E-03 |
|  | *CPD* | carboxypeptidase D | 1.408 | 5.04E-03 |
|  | *HIST1H2BG* | histone cluster 1, H2bg | 1.405 | 4.09E-03 |
|  | *NEURL2* | neuralized E3 ubiquitin protein ligase 2 | 1.402 | 1.95E-03 |
|  | *VMP1* | vacuole membrane protein 1 | 1.383 | 6.16E-04 |
|  | *STEAP3* | STEAP family member 3, metalloreductase | 1.376 | 3.72E-03 |
|  | *DTX2* | deltex 2, E3 ubiquitin ligase | 1.365 | 9.80E-05 |
|  | *PHF21A* | PHD finger protein 21A | 1.362 | 3.56E-03 |
|  | *JDP2* | Jun dimerization protein 2 | 1.353 | 7.15E-03 |
|  | *CLK1* | CDC-like kinase 1 | 1.348 | 4.43E-03 |
|  | *PIM1* | Pim-1 proto-oncogene, serine/threonine kinase | 1.345 | 7.62E-03 |
|  | *OSMR* | oncostatin M receptor | 1.337 | 1.07E-03 |
|  | *CEACAM1* | carcinoembryonic antigen-related cell adhesion molecule 1 (biliary glycoprotein) | 1.324 | 1.94E-03 |
|  | *PGS1* | phosphatidylglycerophosphate synthase 1 | 1.321 | 2.27E-03 |
|  | *NDST2* | N-deacetylase/N-sulfotransferase (heparan glucosaminyl) 2 | 1.311 | 5.42E-04 |
|  | *BID* | BH3 interacting domain death agonist | 1.307 | 5.18E-03 |
|  | *NOL12* | nucleolar protein 12 | 1.303 | 1.59E-03 |
|  | *CCT6A* | chaperonin containing TCP1, subunit 6A (zeta 1) | 1.279 | 1.54E-03 |
|  | *PGR* | progesterone receptor | 1.278 | 3.63E-03 |
|  | *ATL3* | atlastin GTPase 3 | 1.272 | 1.41E-03 |
|  | *B9D2* | B9 protein domain 2 | 1.263 | 2.20E-03 |
|  | *SEMA3F* | sema domain, immunoglobulin domain (Ig), short basic domain, secreted, (semaphorin) 3F | 1.261 | 1.72E-03 |
|  | *BIRC2* | baculoviral IAP repeat containing 2 | 1.256 | 8.70E-03 |
|  | *IMP4* | IMP4, U3 small nucleolar ribonucleoprotein | 1.254 | 9.86E-04 |
|  | *IFNGR1* | interferon gamma receptor 1 | 1.253 | 2.87E-03 |
|  | *LGALS8* | lectin, galactoside-binding, soluble, 8 | 1.251 | 5.19E-03 |
|  | *AGAP3* | ArfGAP with GTPase domain, ankyrin repeat and PH domain 3 | 1.25 | 4.34E-03 |
|  | *CD300LF* | CD300 molecule-like family member f | 1.244 | 8.93E-03 |
|  | *CSGALNACT2* | chondroitin sulfate N-acetylgalactosaminyltransferase 2 | 1.241 | 9.44E-03 |
|  | *HARS* | histidyl-tRNA synthetase | 1.238 | 5.53E-03 |
|  | *HIST2H2BF* | histone cluster 2, H2bf | 1.235 | 1.97E-03 |
|  | *KLHL28* | kelch-like family member 28 | 1.234 | 1.43E-03 |
|  | *AK1* | adenylate kinase 1 | 1.233 | 1.52E-03 |
|  | *TP53BP1* | tumor protein p53 binding protein 1 | 1.232 | 6.39E-03 |
|  | *CMSS1* | cms1 ribosomal small subunit homolog (yeast) | 1.231 | 7.60E-03 |
|  | *UBQLN1* | ubiquilin 1 | 1.228 | 4.85E-03 |
|  | *FGA* | fibrinogen alpha chain | 1.223 | 2.90E-03 |
|  | *RAP1GDS1* | RAP1, GTP-GDP dissociation stimulator 1 | 1.213 | 2.48E-03 |
|  | *PUS3* | pseudouridylate synthase 3 | 1.209 | 2.70E-05 |
|  | *FLCN* | folliculin | 1.207 | 1.84E-03 |
|  | *BAZ1A* | bromodomain adjacent to zinc finger domain, 1A | 1.204 | 8.83E-03 |
|  | *HIST1H3D* | histone cluster 1, H3d | 1.201 | 8.50E-03 |
|  | *FOSB* | FBJ murine osteosarcoma viral oncogene homolog B | -2.652 | 8.73E-03 |
|  | *TACSTD2* | tumor-associated calcium signal transducer 2 | -2.334 | 9.47E-03 |
|  | *MGAT3* | mannosyl (beta-1,4-)-glycoprotein beta-1,4-N-acetylglucosaminyltransferase | -1.702 | 2.04E-03 |
|  | *SASH1* | SAM and SH3 domain containing 1 | -1.536 | 6.36E-03 |
|  | *CYB561D1* | cytochrome b561 family, member D1 | -1.467 | 1.96E-03 |
|  | *FAM53B* | family with sequence similarity 53, member B | -1.448 | 5.53E-03 |
|  | *CRYZ* | crystallin, zeta (quinone reductase) | -1.438 | 9.11E-03 |
|  | *C22orf29* | chromosome 22 open reading frame 29 | -1.436 | 2.44E-03 |
|  | *ZHX3* | zinc fingers and homeoboxes 3 | -1.428 | 2.39E-04 |
|  | *VTCN1* | V-set domain containing T cell activation inhibitor 1 | -1.412 | 1.12E-03 |
|  | *ARRB1* | arrestin, beta 1 | -1.39 | 8.54E-03 |
|  | *CCDC146* | coiled-coil domain containing 146 | -1.385 | 5.73E-03 |
|  | *AMT* | aminomethyltransferase | -1.374 | 9.19E-03 |
|  | *WASF2* | WAS protein family, member 2 | -1.373 | 2.56E-03 |
|  | *SBF1* | SET binding factor 1 | -1.37 | 3.01E-03 |
|  | *TRIM25* | tripartite motif containing 25 | -1.359 | 7.64E-03 |
|  | *GID8* | GID complex subunit 8 | -1.332 | 2.03E-03 |
|  | *NT5DC3* | 5'-nucleotidase domain containing 3 | -1.327 | 6.70E-03 |
|  | *SNORA28* | small nucleolar RNA, H/ACA box 28 | -1.31 | 3.24E-03 |
|  | *CREB3L2* | cAMP responsive element binding protein 3-like 2 | -1.289 | 9.95E-03 |
|  | *FAM168B* | family with sequence similarity 168, member B | -1.284 | 8.60E-03 |
|  | *SMIM19* | small integral membrane protein 19 | -1.28 | 6.03E-03 |
|  | *CUTA* | cutA divalent cation tolerance homolog (E. coli) | -1.274 | 6.19E-03 |
|  | *FOXK1* | forkhead box K1 | -1.265 | 8.53E-03 |
|  | *MPC1* | mitochondrial pyruvate carrier 1 | -1.263 | 9.96E-03 |
|  | *GRB2* | growth factor receptor-bound protein 2 | -1.246 | 5.81E-03 |
|  | *PARVA* | parvin, alpha | -1.245 | 2.10E-03 |
|  | *GRAMD4* | GRAM domain containing 4 | -1.236 | 7.82E-03 |
|  | *CD99* | CD99 molecule | -1.228 | 4.33E-03 |
|  | *BTBD7* | BTB (POZ) domain containing 7 | -1.224 | 5.16E-03 |
|  | *SERPINB9* | serpin peptidase inhibitor, clade B (ovalbumin), member 9 | -1.221 | 9.17E-03 |
|  | *YPEL5* | yippee-like 5 (Drosophila) | -1.208 | 9.67E-03 |
|  | *ZZEF1* | zinc finger, ZZ-type with EF-hand domain 1 | -1.207 | 3.79E-03 |

**Supplementary Table III – Enriched KEGG pathways in TL vs. TNL**

|  | **Name of KEGG pathway** | ***p* value** | **Genes** | **No. of significant genes** | **% of significant genes** |
| --- | --- | --- | --- | --- | --- |
| **Up** | TNF signaling pathway | 8.45E-06 | *IL6, MAP3K8, NFKBIA, PTGS2, SOCS3, TNFAIP3* | 6 | 5.8 |
|  | Legionellosis | 1.15E-04 | *CLK1, HSPA1B, IL6, NFKBIA* | 4 | 7.5 |
|  | Measles | 1.94E-03 | *HSPA1B, IL6, NFKBIA, TNFAIP3* | 4 | 3.6 |
|  | NOD-like receptor signaling pathway | 2.11E-03 | *IL6, NFKBIA, TNFAIP3* | 3 | 5.7 |
|  | Osteoclast differentiation | 2.42E-03 | *FOSL1, FOSL2, NFKBIA, SOCS3* | 4 | 3.4 |
|  | HTLV-I infection | 5.20E-03 | *ATF3, ETS1, FOSL1, IL6, NFKBIA* | 5 | 2.1 |
|  | Influenza A | 5.61E-03 | *HSPA1B, IL6, NFKBIA, SOCS3* | 4 | 2.7 |
|  | NF-kappa B signaling pathway | 8.54E-03 | *NFKBIA, PTGS2, TNFAIP3* | 3 | 3.4 |
|  | Toll-like receptor signaling pathway | 8.54E-03 | *IL6, MAP3K8, NFKBIA* | 3 | 3.4 |
|  | FoxO signaling pathway | 1.82E-02 | *CDKN2D, IL6, PLK3* | 3 | 2.6 |
|  | Spliceosome | 2.22E-02 | *EIF4A3, HSPA1B, NHP2L1* | 3 | 2.4 |
|  | Cytosolic DNA-sensing pathway | 2.29E-02 | *IL6, NFKBIA* | 2 | 4.2 |
|  | Adipocytokine signaling pathway | 3.26E-02 | *NFKBIA, SOCS3* | 2 | 3.4 |
|  | Leishmaniasis | 4.01E-02 | *NFKBIA, PTGS2* | 2 | 3.1 |
|  | Herpes simplex infection | 4.26E-02 | *IL6, NFKBIA, SOCS3* | 3 | 1.9 |
|  | MicroRNAs in cancer | 4.47E-02 | *DDIT4, MIR21, PTGS2* | 3 | 1.8 |
| **Down** | Oxidative phosphorylation | 3.72E-02 | *LHPP, NDUFS8* | 2 | 1.8 |

**Supplementary Table IV – Enriched KEGG pathways in PTL vs. TL**

|  | **Name of KEGG pathway** | ***p* value** | **Genes** | **No. of signficant genes** | **% of significant genes** |
| --- | --- | --- | --- | --- | --- |
| **Up** | Complement and coagulation cascades | 1.08E-03 | *FGA, FGB, FGG, PLAT* | 4 | 7.5 |
|  | Platelet activation | 1.83E-02 | *FGA, FGB, FGG, TLN1* | 4 | 3.4 |
|  | Phagosome | 2.91E-02 | *ATP6V0B, ATP6V1B2, MARCO, TLR4* | 4 | 3 |
|  | Rheumatoid arthritis | 3.19E-02 | *ATP6V0B, ATP6V1B2, TLR4* | 3 | 3.8 |
|  | Butirosin and neomycin biosynthesis | 4.28E-02 | *HK3* | 1 | 20 |
| **Down** | mRNA surveillance pathway | 1.11E-02 | *UPF3A, WDR33* | 2 | 2.5 |
|  | HIF-1 signaling pathway | 1.52E-02 | *CDKN1B, RPS6* | 2 | 2.2 |
|  | Insulin signaling pathway | 2.54E-02 | *PPP1R3B, RPS6* | 2 | 1.6 |

**Supplementary Table V – Enriched KEGG pathways in PTL vs. PTNL**

|  | **Name of KEGG pathway** | ***p* value** | **Genes** | **No. of signficant genes** | **% of significant genes** |
| --- | --- | --- | --- | --- | --- |
| **Up** | Cytokine-cytokine receptor interaction | 1.07E-03 | *CCR7, CSF3R, CXCL8, IFNGR1, IL7R, OSMR, TNFRSF6B, TNFSF14* | 8 | 3.9 |
|  | Complement and coagulation cascades | 2.11E-03 | *FGA, FGB, FGG, SERPINA1* | 4 | 7.5 |
|  | Legionellosis | 2.11E-03 | *CLK1, CXCL8, HSPA1A, HSPA1B* | 4 | 7.5 |
|  | Jak-STAT signaling pathway | 7.84E-03 | *CSF3R, IFNGR1, IL7R, OSMR, PIM1* | 5 | 4.2 |
|  | Nicotinate and nicotinamide metabolism | 2.54E-02 | *NAMPT, NNMT* | 2 | 8.3 |
|  | Toxoplasmosis | 2.62E-02 | *BIRC2, HSPA1A, HSPA1B, IFNGR1* | 4 | 3.7 |
|  | Hematopoietic cell lineage | 4.27E-02 | *CSF3R, IL7R, TFRC* | 3 | 4 |
|  | Apoptosis | 4.70E-02 | *BID, BIRC2, IRAK3* | 3 | 3.8 |
|  | Endocytosis | 4.93E-02 | *AGAP3, HSPA1A, HSPA1B, PLD1, TFRC* | 5 | 2.6 |
| **Down** | Alcoholism | 2.38E-03 | *CREB3L2, FOSB, GRB2* | 3 | 2.4 |
|  | Cocaine addiction | 3.02E-03 | *CREB3L2, FOSB* | 2 | 5.4 |
|  | Amphetamine addiction | 5.04E-03 | *CREB3L2, FOSB* | 2 | 4.2 |
|  | Prostate cancer | 1.42E-02 | *CREB3L2, GRB2* | 2 | 2.4 |
|  | Choline metabolism in cancer | 1.56E-02 | *GRB2, WASF2* | 2 | 2.3 |
|  | Estrogen signaling pathway | 1.63E-02 | *CREB3L2, GRB2* | 2 | 2.3 |
|  | Cell adhesion molecules (CAMs) | 2.74E-02 | *CD99, VTCN1* | 2 | 1.7 |
|  | Osteoclast differentiation | 2.82E-02 | *FOSB, GRB2* | 2 | 1.7 |
|  | Hepatitis B | 3.14E-02 | *CREB3L2, GRB2* | 2 | 1.6 |
|  | Phototransduction | 3.36E-02 | *ARRB1* | 1 | 6.7 |
|  | One carbon pool by folate | 3.80E-02 | *AMT* | 1 | 5.9 |
|  | Dorso-ventral axis formation | 4.89E-02 | *GRB2* | 1 | 4.5 |
